# Dissecting the Mechanism of Intracellular *Mycobacterium smegmatis* Growth Inhibition by Platelet Activating Factor C-16

**DOI:** 10.3389/fmicb.2020.01046

**Published:** 2020-06-10

**Authors:** Muhammad Suleman Riaz, Anuvinder Kaur, Suha Nadim Shwayat, Shahriar Behboudi, Uday Kishore, Ansar Ahmed Pathan

**Affiliations:** ^1^Division of Biosciences, College of Health and Life Sciences, Brunel University London, Uxbridge, United Kingdom; ^2^Department of Biotechnology, Abdul Wali Khan University, Mardan, Pakistan; ^3^The Pirbright Institute, Woking, United Kingdom; ^4^School of Veterinary Medicine, Faculty of Health and Medical Sciences, University of Surrey, Guildford, United Kingdom

**Keywords:** platelet activating factor, PAF analogs, *Mycobacterium tuberculosis*, *Mycobacterium smegmatis*, THP-1 cells, intracellular mycobacterial growth

## Abstract

*Mycobacterium tuberculosis* (*M.tb*) infection results in approximately 1.3 million human deaths each year. *M.tb* resides primarily inside macrophages, and maintains persistent infection. In response to infection and inflammation, platelet activating factor C-16 (PAF C-16), a phospholipid compound, is released by various cells including neutophils and monocytes. We have recently shown that PAF C-16 can directly inhibit the growth of two representative non-pathogenic mycobacteria, *Mycobacterium bovis BCG* and *Mycobacterium smegmatis* (*M. smegmatis*), by damaging the bacterial cell membrane. Here, we have examined the effect of PAF C-16 on *M. smegmatis* residing within macrophages, and identified mechanisms involved in their growth inhibitory function. Our results demonstrated that exogenous PAF C-16 inhibited the growth of *M. smegmatis* inside phagocytic cells of monocytic cell line, THP-1; this effect was partially blocked by PAF receptor antagonists, suggesting the involvement of PAF receptor-mediated signaling pathways. Arachidonic acid, a downstream metabolite of PAF C-16 signaling pathway, directly inhibited the growth of *M. smegmatis in vitro*. Moreover, the inhibition of phospholipase C and phospholipase A_2_ activities, involved in PAF C-16 signaling pathway, increased survival of intracellular *M. smegmatis*. Interestingly, we also observed that inhibition of inducible nitric oxide synthase (iNOS) enzyme and antibody-mediated neutralization of TNF-α partially mitigated the intracellular growth inhibitory effect of PAF C-16. Use of a number of PAF C-16 structural analogs, including Lyso-PAF, 2-O-methyl PAF, PAF C-18 and Hexanolamino PAF, revealed that the presence of acetyl group (CH_3_CO) at *sn*-2 position of the glycerol backbone of PAF is important for the intracellular growth inhibition activity against *M. smegmatis*. Taken together, these results suggest that exogenous PAF C-16 treatment inhibits intracellular *M. smegmatis* growth, at least partially, in a nitric oxide and TNF-α dependent manner.

## Introduction

*Mycobacterium tuberculosis* (*M.tb*) belongs to the acid-fast group of bacteria and causes an infectious disease in humans, known as Tuberculosis (TB), which mostly affects the respiratory system. Approximately 1.3 million people died as a result of *M.tb* infections in 2017, which is the highest number of human mortalities caused by any single bacterial pathogen (World Health Organization [WHO], 2018). Nearly 10 million new cases of *M.tb* infection were reported worldwide during 2017, the majority of infected population belongs to developing countries such as India, Indonesia, China, the Philippines, Pakistan, and Nigeria (World Health Organization [WHO], 2018). It is estimated that about one-third of the world’s population are latently infected with *M.tb* (Flynn and Chan, 2001). The latently infected individuals show no evidence of active disease due to the containment of the pathogen by the host immune system. Over the course of time, this latent *M.tb* infection can reactivate into active disease, and thus, provides a vast reservoir for the spread of *M.tb* infection.

New challenges such as HIV-1 and *M.tb* co-infections in TB patients, multi-drug resistant (MDR), and extensively drug resistant (XDR) strains of *M.tb* have also emerged recently. During HIV-1 and *M.tb* co-infection, the virus infects the CD4^+^T cells, which are the most important immune cells involved in controlling *M.tb* infection (Daley et al., 1992). The HIV infection, thus, not only predisposes the patients to new *M.tb* infections, but also increases the chances of reactivation of latent TB due to host’s immunocompromised status. The emergence of MDR and XDR strains of *M.tb* is a global challenge in treating TB patients as the patients fail to respond to multiple anti-TB drugs, and hence, can act as a reservoir for the spread of drug-resistant *M.tb* strains (Espinal et al., 2000; Ormerod, 2005; Liu et al., 2011). The current vaccine against TB consists of attenuated *Mycobacterium bovis* strain, Bacillus Calmette-Guéerin (BCG), which is almost 100 years old with variable efficacy, and is not effective in the adult population (Sepulveda et al., 1992; Colditz et al., 1994; Aronson et al., 2004; Lahey and von Reyn, 2016). Therefore, to control the global menace of TB, novel interventions are required on the therapeutic and preventive fronts.

*Mycobacterium tuberculosis* infection of the host evokes localized inflammation in the lungs, resulting in the migration of different immune cells and the leakage of plasma proteins and non-proteinaceous factors at the site of infection due to changes in vascular permeability (Sherwood and Toliver-Kinsky, 2004; Amaral et al., 2016). In addition, phospholipids, such as PAF C-16 and proteins such as C1q, are synthesized by the host’s immune cells, which are present at the site of infection (Camussi et al., 1987; Kaul and Loos, 1995). These host factors are likely to come in direct contact with the bacterial pathogen and immune cells, and thus, may modulate the outcome of the infection. The effects of the majority of these host factors on *M.tb* growth, intracellular as well as extracellular, are either poorly understood or completely unknown.

Platelet activating factor (PAF) is a phospholipid compound that is involved in a number of important biological processes in mammals, including platelet aggregation (Chesney et al., 1982), inflammation and allergy (Henderson et al., 2000). Chemically, PAF is 1-*O*-alkyl-2-acetyl-*sn*-glyceryl-3-phosphocholine (Demopoulos et al., 1979). The most common form of naturally produced PAF in humans contains a 16-carbon chain attached at the *sn*-1 position via ether linkage, and is known as PAF C-16 (Clay et al., 1984). PAF C-16 is normally present in picogram per milliliter concentrations in human serum; however, its production is increased during inflammation, allergic reactions and in newly diagnosed TB patients, particularly in cavitary form of pulmonary TB (Kaminskaia and Aladysheva, 1995; Vadas et al., 2013). Moreover, neutrophils from TB patients, when stimulated with BCG *in vitro*, produced three times more PAF C-16 than control (Kaminskaia and Aladysheva, 1995). A variety of cell types including platelets (Alam et al., 1983), monocytes/macrophages (Leaver et al., 1990; Yagnik, 2014), neutrophils (Biffl et al., 1996), endothelial cells (Bussolino et al., 1986), and mast cells (Schleimer et al., 1986) are able to produce PAF C-16 upon stimulation. PAF C-16 binds to a specific G-protein coupled receptor, PAF receptor (PAFR), on the target cells (Ishii et al., 2002). PAF-PAFR engagement activates downstream signaling pathways, including the activation of phospholipases (PLC and PLA_2_), kinases such as protein tyrosine kinase and protein kinase C as well as the production of cytokines such as TNF-α, IL-1α and prostaglandins (Ishii and Shimizu, 2000; Honda et al., 2002).

It has been shown that PAF C-16 possesses direct growth inhibition activity against mycobacteria (*M. smegmatis* and *M. bovis* BCG) (Riaz et al., 2018) and a number of Gram-positive bacteria (Steel et al., 2002) by causing damage to the cell membrane. Exogenous PAF C-16 has also been shown to inhibit the growth of intracellular pathogenic protozoans such as Leishmania and Trypanosoma inside human and mouse macrophages by causing the production of reactive oxygen and nitrogen species (Aliberti et al., 1999; Lonardoni et al., 2000; Borges et al., 2017). Similarly, administration of exogenous PAF C-16 in mice, infected with lethal doses of *Candida albicans*, reduced the number of pathogens and improved the survival via production of NO and TNF-α (Im et al., 1997; Kim et al., 2008).

In the current study, we examined PAF C-16 and its structural analogs *in vitro* for their effect on the growth of *M. smegmatis* (as a model for *M.tb*) inside human monocytes derived THP-1 cells. Furthermore, the underlying mechanisms of PAF C-16 induced growth inhibition of intracellular *M. smegmatis* were also investigated.

## Experimental Procedures

### Chemicals

**FIGURE 1:**
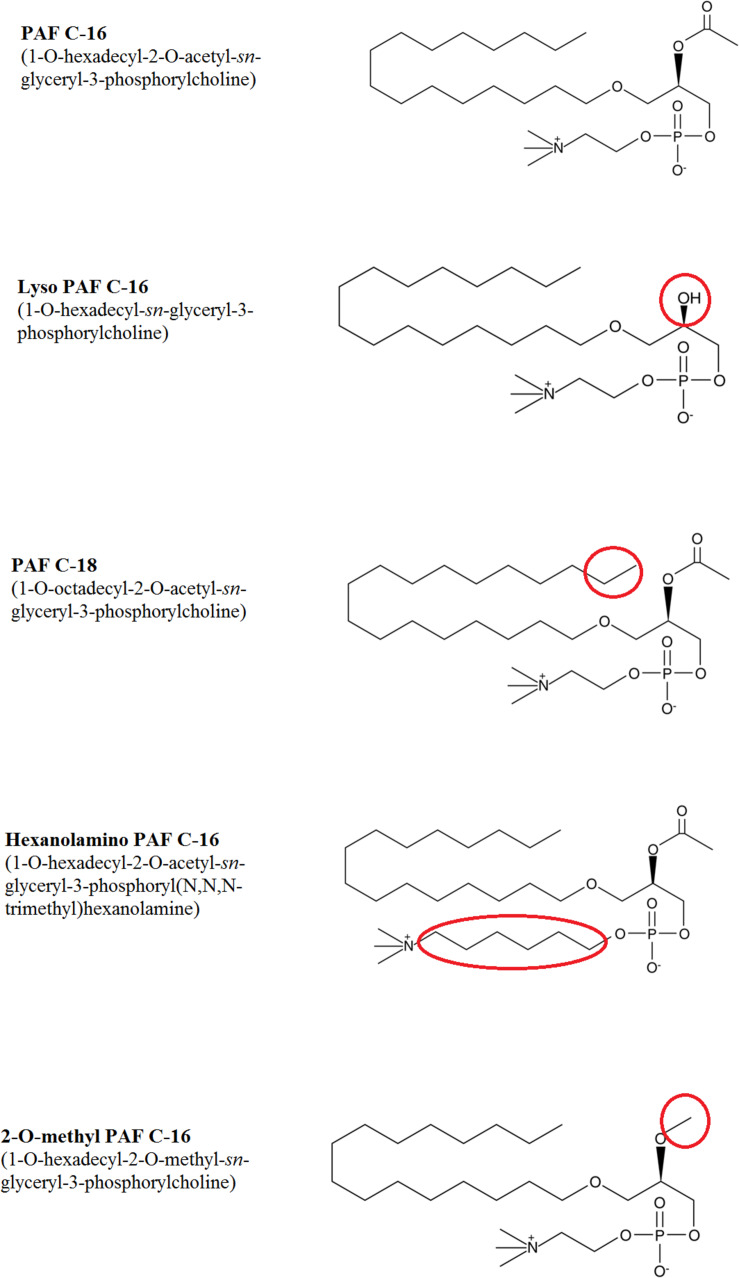
Chemical structures of PAF C-16 and its various analogs tested against *M. smegmatis*. The changes in structure for different analogs are highlighted in red circles.

PAF C-16 (1-*O*-hexadecyl-2-*O*-acetyl-*sn*-glyceryl-3-phosphoryl choline) and different PAF C-16 structural analogs, including Lyso-PAF (1-*O*-hexadecyl-2- hydroxy-*sn*-glyceryl-3-phosphoryl choline), PAF C-18 (1-*O*-octadecyl-2-*O*-acetyl-*sn*-glyceryl- 3-phosphorylcholine), Hexanolamino PAF [1-*O*-hexadecyl-2-*O*- acetyl-*sn*-glyceryl-3-phosphoryl (*N,N,N*-trimethyl) hexanol amine], 2-*O*-methyl PAF (1-*O*-hexadecyl-2-*O*-methyl-*sn*-glyceryl-3-phosphorylcholine), as shown in Figure 1, along with ABT-491 (1-(*N,N*-Dimethylcarbamoyl)-4-ethynyl-3-(3-fluoro-4-((1H-2-methylimidazo[4,5-c]pyridin-1-yl)methyl) benzoyl)-indole, HCl), U-73122 (1-[6-[((17β)-3-Methoxyestra-1,3,5[10]-trien-17-yl)amino]hexyl]-1H-pyrrole-2,5-dione) and arachidonic acid were purchased from Cayman Chemical Company, United States. WEB-2086 (3-[4-(2-Chlorophenyl)-9-methyl-6H-thieno[3,2-f][1,2,4]triazolo[4,3-a][1,4]diazepin-2-yl]-1-(4-morpholinyl)-1-propanone), benzenesulfonamide and aminoguanidine hemisulfate were purchased from Sigma-Aldrich Company, United States. All the chemicals used in different experiments were of analytical grade.

Stock solutions of PAF C-16, PAF C-16 structural analogs, WEB-2086, U-73122 and arachidonic acid were prepared in ethanol according to the manufacturer’s protocol. ABT-491 and aminoguanidine hemisulfate stock solutions were prepared using distilled water; while the stock solution of benzenesulfonamide was prepared in methanol. Appropriate solvent controls were included in all the experiments involving PAF C-16, its structural analogs or any other chemical compound used.

### Culturing of *M. smegmatis*

*M. smegmatis* (mc^2^ 155) were grown in Luria-Bertani (LB) broth (Lennox; Sigma Aldrich) containing 50 μg/ml carbenicillin (Fisher Scientific, United Kingdom), 0.15% (v/v) glycerol (Fisher Scientific, United Kingdom) and 0.10% (v/v) Tween-80 (Fisher Scientific, United Kingdom) in a shaking incubator at 37°C until the O.D_600 nm_ reached 0.8–0.9. The number of *M. smegmatis* colony forming units (CFUs) per μl was determined by plating different dilutions of the bacterial stock on LB agar plates in triplicates and counting the number of CFUs after incubation at 37°C for 72 h.

The LB agar plates for growing *M. smegmatis* colonies were prepared by dissolving tryptone (10 g) (Fisher Scientific, United Kingdom), yeast extract (5 g) (Fisher Scientific, United Kingdom), sodium chloride (0.5 g) (Fisher Scientific, United Kingdom) and agar (15 g) (Fisher Scientific, United Kingdom) in 1 Litre distilled water and autoclaving the media at 121°C for 15 min. About 15–20 ml of melted LB agar was poured in each plate (petri dish) under sterile conditions and the plates were allowed to solidify for 90–120 min before plating *M. smegmatis*.

### THP-1 Cell Culture

THP-1 cells (a human monocytic leukemic cell line; ATCC^®^ TIB-202^TM^), used as the model phagocytic cells, were grown in complete RPMI (cRPMI) medium at 37°C using an incubator with 5% CO_2_ supply. The cRPMI medium was prepared by adding 10% v/v fetal bovine serum (FBS) (HyClone, United Kingdom), 1 mM sodium pyruvate (Sigma Aldrich, United Kingdom), 2 mM L-glutamine (Sigma Aldrich, United Kingdom) and PenStrep (100 U/mL Penicillium and 100 μg/mL Streptomycin) (Sigma Aldrich, United Kingdom) to RPMI-1640 (Sigma Aldrich, United Kingdom). Cells were fed every 3 days by removing half of the culture medium and replacing it with fresh cRPMI; the cell density was kept at about 0.5–0.75 × 10^6^/ml.

### Intracellular Growth Inhibition Assay for *M. smegmatis* Using THP-1 Cells

Intracellular bacterial growth inhibition assays were performed to investigate the effect of different test compounds, including PAF C-16 and PAF structure analogs, on the growth of phagocytosed *M. smegmatis* inside THP-1 cells. THP-1 cells, grown at a density of 0.5–0.75 × 10^6^/ml, were washed twice with plain RPMI medium and adjusted to ∼0.25 × 10^6^ cells/ml in cRPMI without antibiotics. One ml of this cell suspension was added to individual Eppendorf tubes. Approximately 1.25 × 10^6^ *M. smegmatis* (bacteria to THP1 ratio; 5:1) was then added to each tube. These tubes were incubated at 37°C in a CO_2_ incubator for 2 h with intermittent shaking to allow phagocytosis of bacteria.

Pan anti-mouse IgG coated magnetic Dynabeads (Thermo Fisher Scientific, United Kingdom), bound to mouse pan anti-human MHC class I (HLA A, B, and C) antibody W6/32 (BioLegend, United States), were prepared to remove non-phagocytosed *M. smegmatis*. For each sample, 10^6^ Dynabeads were incubated with 1 μg of W6/32 for 90 min on ice to allow the binding of the beads with W6/32 antibody. Later, the beads were washed twice with plain RPMI by applying a Dynal^®^ magnet (Thermo Fisher Scientific, United Kingdom) and this reagent (Dynabeads-W6/32) was resuspended in 25 μl of RPMI. The ‘Dynabeads-W6/32’ were then added to the previously prepared tube, containing THP-1 cells with *M. smegmatis* (beads to cells ratio; 4:1). The Eppendorf tubes were then placed on ice for 45 min with intermittent shaking to allow the binding of W6/32 component of ‘Dynabeads-W6/32’ with MHC Class I molecules present on THP-1 cells. After incubation, extracellular *M. smegmatis* was removed by applying a Dynal^®^ magnet and washing the cells twice with plain RPMI. This was followed by resuspending the cells in 1ml cRPMI without antibiotic. For each experiment, a solvent control and test compounds treated samples were included and the tubes were incubated at 37°C in a CO_2_ incubator for another 24 h. After incubation, Dynal^®^ magnet was applied to the Eppendorf tubes. The ‘Dynabeads-W6/32’attached to THP-1 cells migrated to the side of Eppendorf tube. The supernatant was removed and stored in 15 ml falcon tubes to collect any extracellular bacteria released from dying cells. The THP-1 cells left in the Eppendorf tubes were lysed by adding 1ml of 1% w/v saponin solution in water (Fisher Scientific, United Kingdom) and vortexing the mixture for 15 min. The cell lysate for each condition was transferred to the respective 15 ml falcons already containing 1 ml of bacterial supernatant that was collected earlier. The contents of each falcon tube were mixed by vortexing for 10 s and serial dilutions (10^–1^, 10^–2^, and 10^–3^) were prepared in sterile PBS.

Finally, the bacterial suspensions, at dilutions of 10^–2^ and 10^–3^, were used for plating. 200 μl of bacterial suspension was plated for each experimental condition in triplicates using LB-agar plates. Plates were incubated at 37°C for 72 h, after which the number of bacterial CFUs were counted. A comparison of CFUs between test compound treated plates and the solvent control was done to determine the effect of the test compound on the growth of intracellular *M. smegmatis* inside THP-1 cells.

### Assessing the Effects of PAF Receptor Antagonists on PAF C-16 Induced Intracellular *M. smegmatis* Growth Inhibition

Two PAFR antagonists, ABT-491 and WEB-2086, were used to examine their effects on PAF C-16 induced intracellular *M. smegmatis* growth inhibition. The assays were carried out according to the intracellular growth inhibition assay as described above, with a difference that *M. smegmatis* infected THP-1 cells were initially treated with PAFR antagonists (ABT-491 or WEB-2086, 2 μg/ml each) for 1 h before adding PAF C-16 (1 μg/ml) to the cell culture, the samples were incubated further for 24 h at 37°C in a CO_2_ incubator. Solvent control along with an additional control comprising of *M. smegmatis* infected THP-1 cells treated with PAFR antagonists only, were also included in the experimental design.

### Assessing the Effects of Chemical Inhibitors of PLC, PLA_2_ and iNOS and Anti-TNF-α Antibody on PAF C-16 Induced Intracellular *M. smegmatis* Growth Inhibition

Assays using inhibitors of PLC (U-73122), cPLA_2_ (benzenesulfonamide) and iNOS (aminoguanidine hemisulfate) were also performed similar to the intracellular growth inhibition assay. The only difference was in the treatment step where *M. smegmatis* infected THP-1 cells were first treated with U-73122 (2 μM) (Macmillan and McCarron, 2010), benzenesulfonamide (56 nM) (Farooqui et al., 2006) or aminoguanidine hemisulfate (1 mM) (Nascimento et al., 2002) for 1 h. Subsequently, PAF C-16 (1 μg/ml) was added and the THP-1 cells containing phagocytosed *M. smegmatis* were further incubated for 24 h at 37°C in a CO_2_ incubator. An additional control comprising of *M. smegmatis* infected THP-1 cells treated with U-73122 (2 μM), benzenesulfonamide (56 nM) or aminoguanidine hemisulfate (1 mM), was also included in the respective experimental designs.

Anti-TNF-α neutralizing antibody (BD Biosciences, United States) was used to investigate the role of TNF-α in PAF C-16 induced growth inhibition of intracellular *M. smegmatis*. *M. smegmatis* infected THP-1 cells were treated with 10 μg/ml of mouse anti-human TNF-α antibody, an isotype antibody control (10 μg/ml mouse IgG) or ethanol (PAF C-16 solvent) for 1 h prior to treatment with 1 μg/ml PAF C-16, and the cells were further incubated for another 24 h before cell lysis and plating.

### Direct Growth Inhibition Assay

This assay was carried out as described previously (Riaz et al., 2018), to investigate the direct effect of arachidonic acid on *M. smegmatis* growth. Briefly, the diluted stock of *M. smegmatis* (2.5 × 10^4^) in suspensions of 1ml LB broth was exposed to a range of concentrations of arachidonic acid for 2 h at 37°C with mixing every 15 min. Appropriate solvent control (10 μl/ml) for the test compound was included. After incubation, 200 μl of bacterial suspensions from test and control tubes were seeded on LB agar plates in triplicate and the plates were incubated at 37°C for 72 h. Colony counting method was used to detect the direct growth inhibitory effects of the test compounds.

### Statistical Analysis

At least three independent experiments were performed. The data were presented as box plots of medians (25th-75th percentile), and the solvent treated samples (control) were considered 100% bacterial survival. Statistical analysis was performed using GraphPad Prism^®^ software (Version 5.01) to determine the level of significance (*p*-value). For intracellular growth inhibition assays, *p*-value was determined by applying non-parametric multiple comparison Kruskal–Wallis test on ranks and any two datasets were compared using Dunn’s *post hoc* test. For comparison of two particular datasets, non-parametric Mann–Whitney test was used. A *p*-value of less than or equal to 0.05 (*p* ≤ 0.05) was considered to be significant. On the graphs, *p* ≤ 0.05 was denoted by ^∗^, *p* ≤ 0.01 by ^∗∗^, and *p* ≤ 0.001 by ^∗∗∗^.

## Results

### PAF C-16 Suppresses *M. smegmatis* Growth Inside THP-1 Cells

**FIGURE 2:**
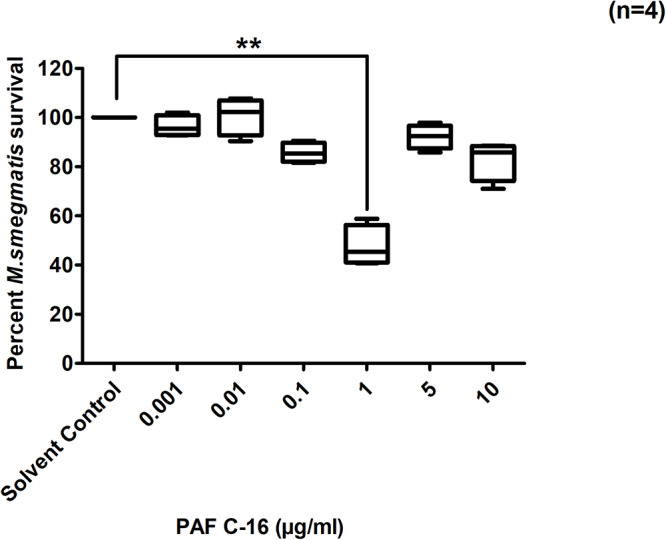
Effect of PAF C-16 treatment on the growth of intracellular *M. smegmatis*. *M. smegmatis* infected THP-1 cell were treated for 24 h with either solvent control (10 μl ethanol/ml) or indicated concentrations of PAF C-16, before lysis and plating. The data shown as box plots represent median, interquartile and minimal and maximal values for four individual experiments performed in triplicates. The data is expressed in terms of percentage, where solvent control is considered as 100% survival. Level of significance was calculated by applying multiple comparison non-parametric Kruskal–Wallis test on ranks (*p* = 0.0012) and any two data sets were compared using *post hoc* Dunn’s multiple comparison, where PAF C-16 (1 μg/ml) vs solvent control was found to be significant, **p ≤ 0.01.

Intracellular growth inhibition assays were performed by treating *M. smegmatis* infected THP-1 cells with PAF C-16 at concentrations ranging between 0.001–10 μg/ml (1.90 nM–19.09 μM) for 24 h and the resulting growth inhibitory effect was determined by counting the number of surviving CFUs on LB agar plates. PAF C-16, at a concentration of 1 μg/ml (1.90 μM), on average inhibited the growth of intracellular *M. smegmatis* by 49% (*n* = 4, independent experiments), compared with the solvent control (10 μl ethanol/ml cell suspension) (*p* ≤ 0.01) (Figure 2). Treatment of *M. smegmatis* infected THP-1 cells with PAF C-16 at concentrations higher or lower than 1 μg/ml [0.001 μg (1.90 nM), 0.01 μg (19.09 nM), 0.1 μg (190.9 nM), 5 μg (9.5 μM), and 10 μg/ml (19.09 μM)] for 24 h showed no significant inhibitory effect on the growth of intracellular *M. smegmatis* (Figure 2).

### Acetyl Group at *sn*-2 Position Is Essential for the Inhibitory Effect of PAF C-16 on *M. smegmatis* Growth Inside THP-1 Cells

**FIGURE 3:**
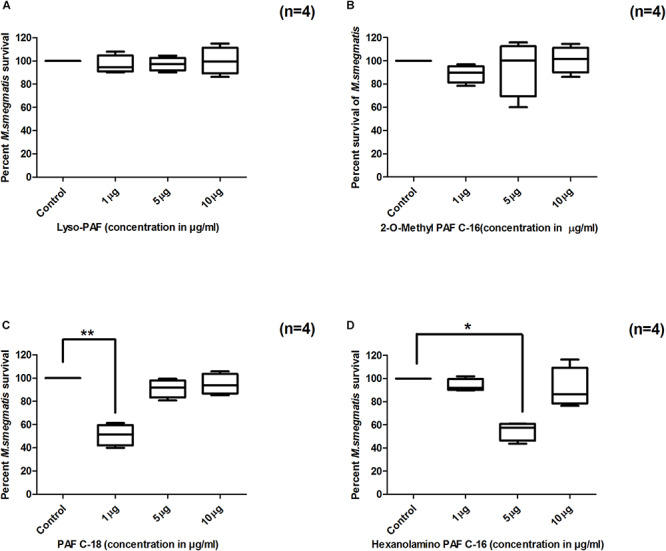
Effect of PAF C-16 structural analogs on the growth of intracellular *M. smegmatis*. *M. smegmatis* infected THP-1 cells were treated for 24 h with either solvent control (10 μl ethanol/ml), Lyso-PAF **(A)**, 2-*O*-methyl PAF **(B)**, PAF C-18 **(C)** or Hexanolamino PAF **(D)** at indicated concentrations, before lysis and plating. The data is expressed in terms of percentage, where solvent control is considered as 100% survival. The data shown as box plots represent median, interquartile and minimal and maximal values for four individual experiments performed in triplicates. Statistically significant differences between tests samples (PAF analogs) versus solvent controls were determined using Kruskal–Wallis test and any two data sets were compared using *post hoc* Dunn’s multiple comparison (**p ≤ 0.01 and *p ≤ 0.05).

To assess the impact of small modifications in the structure of PAF C-16 on its intracellular *M. smegmatis* growth inhibition potential, different PAF C-16 structural analogs, including its precursor form Lyso-PAF, 2-O-methyl PAF, PAF C-18 and Hexanolamino PAF (Figure 1) were tested. The results showed that structural analogs lacking an acetyl group at *sn*-2 position such as Lyso-PAF and 2-O-methyl PAF were unable to inhibit the growth of intracellular *M. smegmatis* at concentrations range of 1, 5, and 10 μg/ml (Figures 3A,B). Both PAF C-18 and Hexanolamino PAF having acetyl group at *sn-2* position were able to inhibit the growth of intracellular *M. smegmatis* at the concentration of 1 and 5 μg/ml, respectively (*p* ≤ 0.01 and *p* ≤ 0.05) (Figures 3C,D), and their intracellular *M. smegmatis* growth inhibition ability was similar to PAF C-16 at 1 μg/ml (∼50% reduction in CFUs).

### PAF Receptor Antagonists Partially Mitigated PAF C-16 Induced Intracellular *M. smegmatis* Growth Inhibition

**FIGURE 4:**
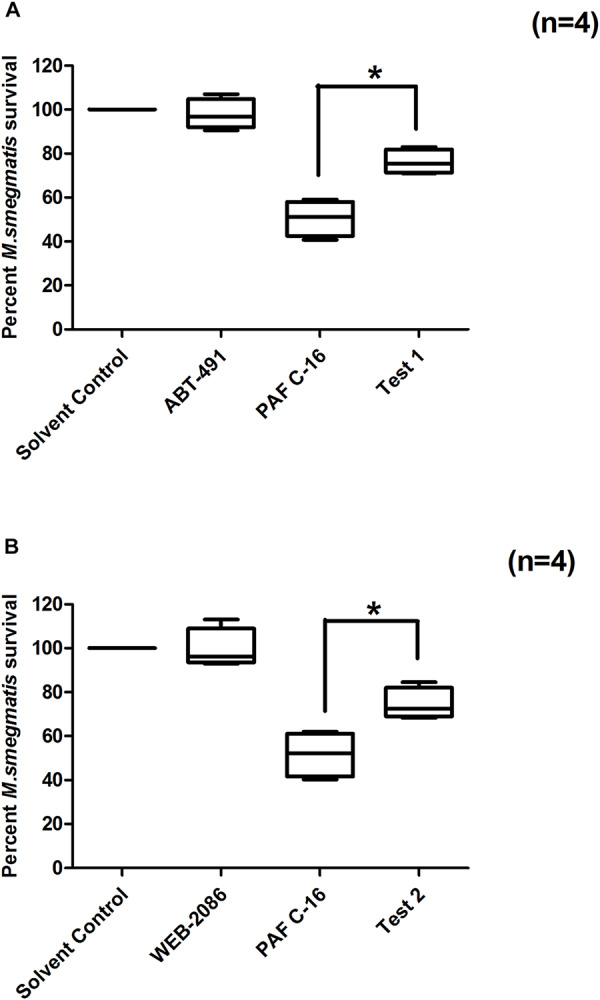
Effect of PAFR antagonists (ABT-491 and WEB-2086) on PAF C-16 induced growth inhibition of intracellular *M. smegmatis*. *M. smegmatis* infected THP-1 samples were treated for 24 h with either solvent control (10 μl ethanol/ml), ABT-491 (2 μg/ml), PAF C-16 (1 μg/ml) and a combination of ABT-491 (2 μg/ml) and PAF C-16 (1 μg/ml) (Test-1) **(A)**, or WEB-2086 (2 μg/ml) and a combination of WEB-2086 (2 μg/ml) and PAF C-16 (1 μg/ml) (Test-2) **(B)**, before lysis and plating. The data is expressed in terms of percentage, where solvent control is taken as 100% survival and different treated conditions are compared to it. The data shown as box plots represent median, interquartile and minimal and maximal values for four individual experiments performed in triplicates. Statistically significant (*p = 0.02) differences were found in the case of both PAFR antagonists with PAF C-16 alone treated samples using Mann–Whitney test.

Two structurally distinct PAFR antagonists, ABT-491 and WEB-2086, were used to investigate their effect on PAF C-16 induced growth inhibition of *M. smegmatis*. Prior treatment of *M. smegmatis* infected THP-1 cells with ABT-491 or WEB-2086 (Test-1 and Test-2, Figure 4) at a concentration of 2 μg/ml partially mitigated the inhibitory effect of PAF C-16 on the growth of intracellular *M. smegmatis*, as indicated by an increase in the of number CFUs (20–24.5%) compared to 1 μg/ml PAF C-16 treated only condition (*n* = 4, independent experiments) (Figures 4A,B). This difference was significant (*p* = 0.02, Mann–Whitney test) for both PAFR antagonists. In the absence of PAF C-16, ABT-491 as well as WEB-2086 showed no effect on the survival of intracellular *M. smegmatis* (Figures 4A,B).

### PLC Inhibitor (U-73122) Partially Overcomes PAF C-16 Induced Intracellular *M. smegmatis* Growth Inhibition

**FIGURE 5:**
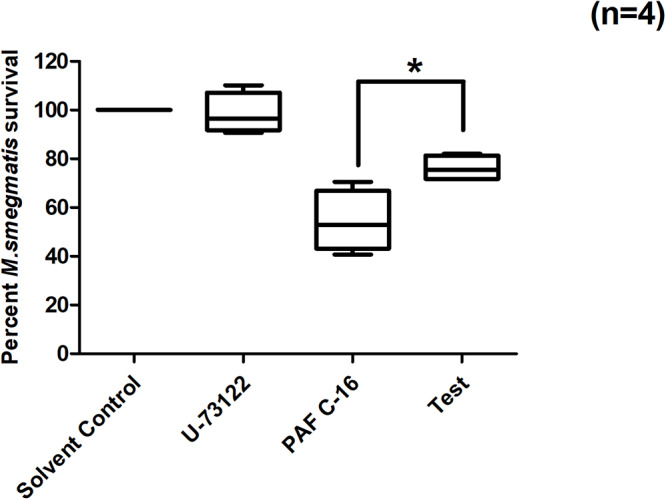
Effect of PLC inhibitor U-73122 on PAF C-16 induced growth inhibition of intracellular *M. smegmatis*. *M. smegmatis* infected THP-1 samples were treated for 24 h with either solvent control (10 μl ethanol/ml), U-73122 (2 μM), PAF C-16 (1 μg/ml) or a combination of U-73122 (2 μM) and PAF C-16 (1 μg/ml) (Test), before lysis and plating. The data is expressed in terms of percentage, where solvent control is taken as 100% survival and different treated conditions are compared to it. The data represent median, interquartile and minimal and maximal values for four individual experiments performed in triplicates. Statistically significant (*p = 0.02) difference was found for Test v 1 μg/ml PAF C-16 using Mann–Whitney test.

PAF C-16 binding to its receptor causes the activation of phospholipase C (PLC). PLC inhibitor, U-73122, was therefore, used to investigate the role of PLC in PAF C-16 induced growth inhibition of intracellular *M. smegmatis*. It was observed that U-73122 at a concentration of 2 μM was effective in partially mitigating the inhibitory effect PAF C-16 on the growth of intracellular *M. smegmatis*. Treatment of *M. smegmatis* infected THP-1 cells with U-73122 along with 1 μg/ml PAF C-16 resulted in ∼25.2% increase in the number of surviving *M. smegmatis* CFUs when compared to 1 μg/ml PAF C-16 only treated condition (Figure 5); this difference was significant (*p* = 0.02, Mann–Whitney test).

### PLA_2_ Inhibitor (Benzenesulfonamide) Partially Overcomes PAF C-16 Induced Intracellular *M. smegmatis* Growth Inhibition

**FIGURE 6:**
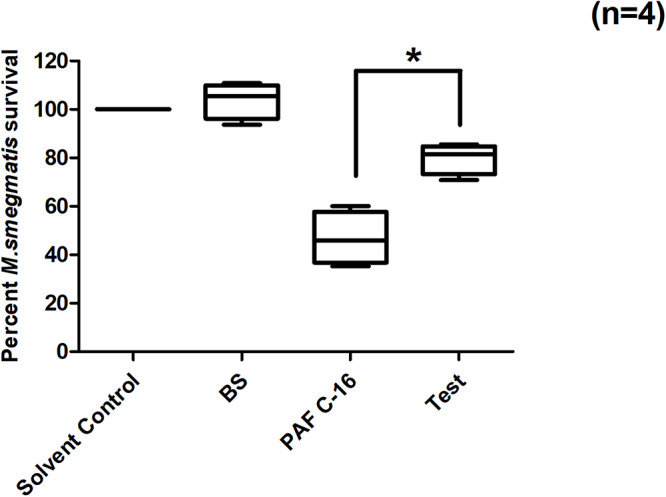
Effect of cPLA_2_ inhibitor Benzenesulfonamide on PAF C-16 induced growth inhibition of intracellular *M. smegmatis*. *M. smegmatis* infected THP-1 samples were treated for 24 h with either solvent control (10 μl ethanol/ml), Benzenesulfonamide (BS, 56 nM), PAF C-16 (1 μg/ml) or a combination of BS (56 nM) and PAF C-16 (1 μg/ml) (Test), before lysis and plating. The data is expressed in terms of percentage, where solvent control is taken as 100% survival and different treated conditions are compared to it. The data represent median, interquartile and minimal and maximal values for four individual experiments performed in triplicates. Statistically significant (*p = 0.02) difference was found for Test v 1 μg/ml PAF C-16 only treated condition using Mann–Whitney test.

PAF C-16 also causes the activation of cytosolic phospholipase A_2_ (cPLA_2_), which leads to the intracellular production of arachidonic acid (AA) and lysophosphatidylcholine. Benzenesulfonamide, a cPLA_2_ inhibitor, was used to investigate the role of cPLA_2_ in PAF C-16 induced growth inhibition of intracellular *M. smegmatis*. *M. smegmatis* infected THP-1 cells were treated with benzenesulfonamide at a concentration of 56nM along with 1 μg/ml PAF C-16 for 24 h. Benzenesulfonamide partially mitigated the inhibitory effect PAF C-16 on the growth of intracellular *M. smegmatis* as indicated by an increase in the number of surviving *M. smegmatis* CFUs. On average, this compound increased the number of *M. smegmatis* CFUs by ∼30.1% when compared to the number of CFUs from 1 μg/ml PAF C-16 only treated condition (Figure 6), and this difference was significant (*p* = 0.02, Mann–Whitney test).

### Arachidonic Acid (AA) Directly Inhibits *M. smegmatis* Growth *in vitro*

**FIGURE 7:**
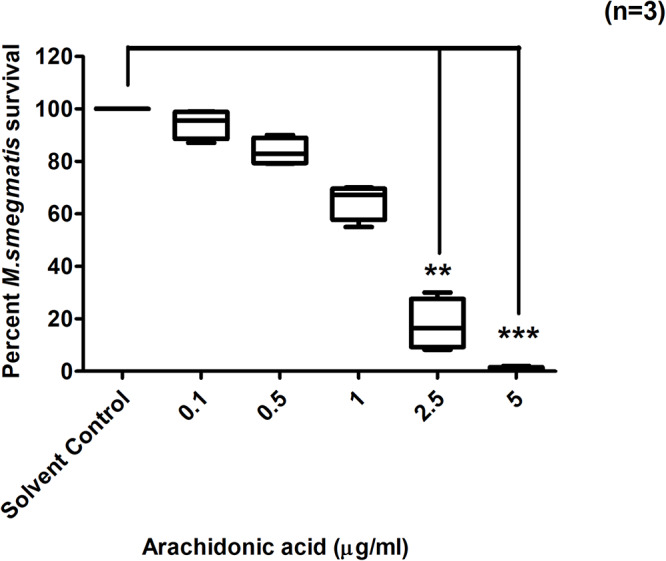
Direct effect of Arachidonic acid on *M. smegmatis* growth *in vitro*. *M. smegmatis* samples were directly treated either with solvent control (10 μl ethanol/ml) or indicated concentrations of arachidonic acid for 2 h before plating. Data is expressed in percentage where solvent control is taken as 100% survival and different arachidonic acid treated conditions are compared to it. The data represent median, interquartile and minimal and maximal values for three individual experiments performed in triplicates. Statistically significant differences between arachidonic acid treated and solvent control samples were estimated using Kruskal–Wallis test (*p* = 0.004) and any two sets of data were compared using *post hoc* Dunn’s multiple comparison. **p ≤ 0.01 and ***p ≤ 0.001.

AA is produced by activated cPLA_2_ from intracellular phospholipids during PAF C-16 signaling pathway. This compound was investigated *in vitro* for its direct effect on the growth of *M. smegmatis* and the results showed dose-dependent growth inhibition of *M. smegmatis* in all the three independent experiments. AA at the concentrations of 5 and 2.5 μg/ml caused a reduction of 99 and 86.4%, respectively in the number of surviving *M. smegmatis* CFUs as compared to solvent control (Figure 7); these results were found to be significant (*p* ≤ 0.001 and *p* ≤ 0.01, respectively) using Dunn’s multiple comparison test. Similarly, other intracellular molecules, Phosphoinositol bisphosphate (PIP_2_) and Diacylglycerol (DAG) produced during PAF C-16 signaling were also tested *in vitro* at the concentrations of 5, 10, 25, 50, and 100 μg/ml, however, they showed no direct inhibitory effect on *M. smegmatis* growth (Supplementary Figures 3, 4).

### Nitric Oxide Synthase Inhibitor (Aminoguanidine Hemisulfate) Partially Overcomes PAF C-16 Induced Growth Inhibition of Intracellular *M. smegmatis*

**FIGURE 8:**
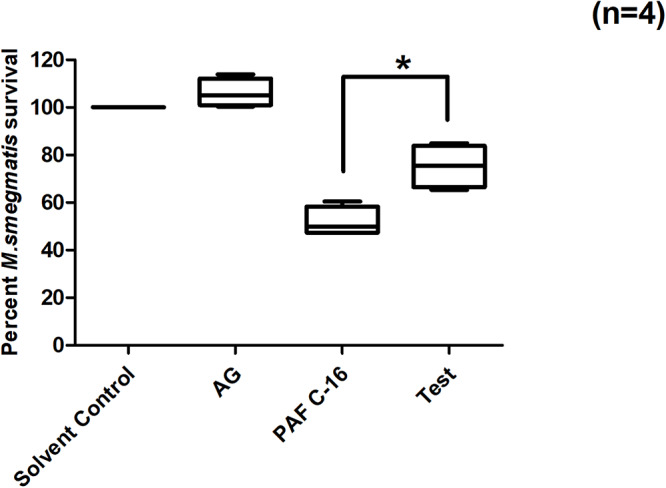
Effect of Aminoguanidine hemisulfate treatment on PAF C-16 induced intracellular *M. smegmatis* growth inhibition. *M. smegmatis* infected THP-1 cells were treated for 24 h either with solvent control (10 μl ethanol/ml), Aminoguanidine hemisulfate (AG, 1 mM), PAF C-16 (1 μg/ml) or a combination of AG (1 mM) and PAF C-16 (1 μg/ml) (Test), before lysis and plating. The data is expressed in terms of percentage, where solvent control is considered as 100% survival and different treatment conditions are compared to it. The data represent median, interquartile and minimal and maximal values for four individual experiments performed in triplicates. Statistically significant (*p = 0.02) difference was found for Test v 1 μg/ml PAF C-16 using Mann–Whitney test.

Treatment of *M. smegmatis* infected THP-1 cells with iNOS inhibitor, aminoguanidine hemisulfate (1 mM), partially mitigated PAF C-16 induced growth inhibition of intracellular *M. smegmatis*. On an average, prior treatment of THP-1 cells having phagocytosed *M. smegmatis* with 1 mM AG increased the number of surviving *M. smegmatis* CFUs by 23.4% when compared to 1 μg/ml PAF C-16 only (Figure 8) and this difference was significant (*p* = 0.02, Mann–Whitney test).

### Anti-TNF-α Antibody Partially Overcomes PAF C-16 Induced Intracellular *M. smegmatis* Growth Inhibition

**FIGURE 9:**
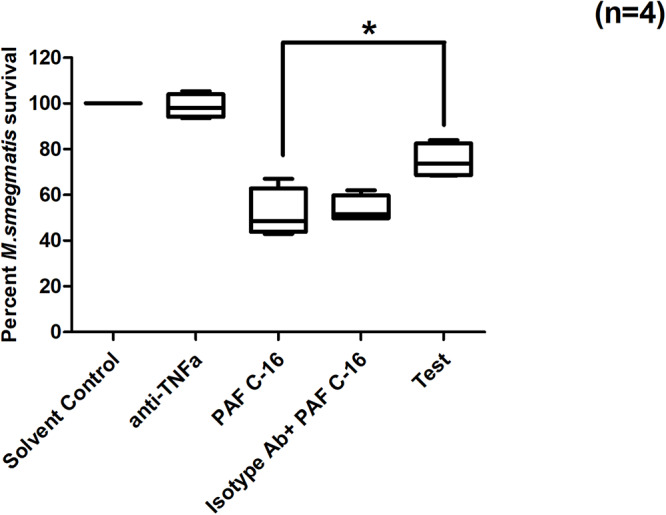
Effect of anti-TNF-α neutralizing antibody on PAF C-16 induced intracellular *M. smegmatis* growth inhibition. *M. smegmatis* infected THP-1 cells were treated for 24 h with solvent control (10 μl ethanol/ml), anti-TNFα antibody (10 μg/ml), PAF C-16 (1 μg/ml), a combination of isotype control antibody (10 μg/ml) with PAF C-16 (1 μg/ml) and anti-TNFα antibody (10 μg/ml) with PAF C-16 (1 μg/ml) (Test), before lysis and plating. The data is expressed in terms of percentage, where solvent control is considered as 100% survival and different treatment conditions are compared to it. The data represent median, interquartile and minimal and maximal values for four individual experiments performed in triplicates. Statistically significant (*p = 0.02) differences were found for ‘Test’ v 1 μg/ml PAF C-16 and ‘Test’ v Isotype control condition using Mann-Whitney test.

To investigate the role of TNF-α on PAF C-16 induced intracellular growth inhibition, *M. smegmatis* infected THP-1 cells were incubated with 10 μg/ml mouse anti-human TNF-α monoclonal antibody (neutralizing antibody) and 1 μg/ml PAF C-16 for 24 h. This antibody partially mitigated the PAF C-16 induced growth inhibition of intracellular *M. smegmatis*, as indicated by 17 and 18% increase in the number of surviving CFUs as compared to the isotype control antibody (10 μg/ml mouse IgG + 1 μg/ml PAF C-16) and 1 μg/ml PAF C-16 only treated conditions respectively (Figure 9) (*p* = 0.02, Mann–Whitney test). Furthermore, neutralizing antibodies specific to IL-6 and IL-10 showed no effect on the intracellular growth inhibitory effect of PAF C-16 (Supplementary Figures 1, 2).

## Discussion

PAF C-16 is a pro-inflammatory phospholipid; its production increases during inflammation (Gawaz et al., 2005) and bacterial infections including *M.tb* infection (Kaminskaia and Aladysheva, 1995; Huseyinov et al., 1999). In this study, the effect of exogenous PAF C-16 was investigated *in vitro* on the growth of intracellular mycobacteria. Phagocytic THP-1 cells infected with *M. smegmatis* were used as a model to investigate the intracellular growth inhibitory effect of PAF C-16. THP-1 cells are human leukemia derived monocytic cells that mimic blood-derived monocytes and are a valuable tool for research because of their homogenous genetic background (Chanput et al., 2014). THP-1 cells have previously been used as an *in vitro* model to study intracellular mycobacteria (Rajavelu and Das, 2007; Fontán et al., 2008; Rohan et al., 2008; Iona et al., 2012). THP-1 cells are usually treated with phorbol 12-myristate 13-acetate (PMA) in order to differentiate them into macrophages (Riendeau and Kornfeld, 2003). PMA treatment of THP-1 cells causes the rearrangement of macrophage-specific kinome that leads to the activation of pro-inflammatory genes, such as IL-1β, IL-8 and TNF-α, making these cells biased toward a more pro-inflammatory phenotype (Richter et al., 2016). Furthermore, IL-1β has been shown to be a potent stimulator of PAF C-16 synthesis in monocyte cell line, U-937 (Vlachogianni et al., 2013). In addition, PAF C-16 is known to modulate the reduced and oxidised Glutathione ratio as well as the redox status of monocytes (Verouti et al., 2011). Therefore, in our study, THP-1 cells, untreated with PMA, were used to provide a microenvironment in which the specific effect of PAF C-16 on the growth of intracellular *M. smegmatis* could be examined.

PAF C-16 can activate monocytes/macrophages by binding to PAFR (Simon et al., 1994) and stimulate the production of inflammatory mediators such as TNF-α, IL-1α and β, reactive oxygen intermediate (ROI) and reactive nitrogen intermediate (RNI) species (Hartung et al., 1983; Bonavida et al., 1989; Poubelle et al., 1991; Muehlmann et al., 2012), and thus may play a protective role during infection. We have recently shown that PAF C-16 and its analogs have direct inhibitory effect on the growth of mycobacteria (Riaz et al., 2018). Therefore, we investigated the effects of these compounds on mycobacterial survival inside phagocytic cells, as these cells have the ability to control mycobacterial infection when they are in an activated state (Leemans et al., 2005). We hypothesized that PAF C-16 and its structurally related compounds may activate macrophages to enhance its intracellular killing ability. In this study, PAF C-16 at an optimal dose of 1 μg/ml was shown to inhibit *M. smegmatis* growth inside THP-1 cells, whereas any deviation (increase or decrease) from this optimal concentration was ineffective. This observation suggests that PAF C-16 acts in a concentration-specific manner. This is consistent with a previous study demonstrating that the treatment of human macrophages with higher concentration (10^–6^ M) of PAF C-16 showed reduced phagocytosis of *Leishmania braziliensis* as compared to cells treated with the lower concentrations (10^–8^ and 10^–10^ M) (Borges et al., 2017). Similarly, human monocytes showed maximum production of ROIs when stimulated with PAF C-16 at 2 × 10^–6^ M; increasing the concentration of PAF C-16 (5 × 10^–6^ M) led to a decrease in ROIs production (Pustynnikov et al., 1991). The fact that PAF C-16 at higher concentrations (5 μg and 10 μg/ml) showed less intracellular growth inhibition than 1 μg/ml in our study, may be due to higher level of PAFR down regulation at these concentrations, as a result of the interaction of PAF C-16 with PAFR. This explanation is supported by the observation made by Chao et al. (1990), who showed that PAF C-16 binding to PAFR results in down regulation of PAFRs in a dose-dependent manner on the surface of rat Kupffer cells.

A number of previous studies have shown that exogenous PAF C-16 can inhibit the growth of intracellular pathogens, including *Leishmania donovani* (Lonardoni et al., 2000), *Leishmania braziliensis* (Borges et al., 2017), *Trypanosoma cruzi* (Aliberti et al., 1999), and *Candida albicans* (Im et al., 1997; Kim et al., 2008) *in vivo* as well as *in vitro*. PAF C-16 induced growth inhibition of these intracellular pathogens was shown to be associated with an enhanced production of ROIs, RNIs, and TNF-α. There is limited information regarding the effect of PAF C-16 on the growth of intracellular mycobacteria. One study investigating the role of endogenous PAF C-16 during *M.tb* infection in mice found that there was no significant difference in mortality between PAFR deficient (PAFR^–/–^) and wild-type control mice when infected with *M.tb* and similar *M.tb* loads were observed in the lungs and liver (Weijer et al., 2003). However, to the best of our knowledge, there is currently no information about the effect of exogenous PAF C-16 or its structural analogs on the growth inhibition of intracellular mycobacteria.

The biological activity of PAF C-16 in eukaryotic cells can be affected by small modifications in its structure (Shigenobu et al., 1985; O’Flaherty et al., 1987; Rose et al., 1990; Stewart and Grigoriadis, 1991). Therefore, the effect of small changes in the structure of PAF C-16 was also investigated on the growth inhibition of intracellular *M. smegmatis* using different PAF C-16 structure analogs. Lyso-PAF is the precursor form of PAF C-16 that contains a hydroxyl group in place of the acetyl group at *sn*-2 position of the glycerol backbone. The enzyme platelet-activating factor acetylhydrolase (PAF-AH) tightly regulates the level of active PAF C-16 in the body and converts excess PAF C-16 into Lyso-PAF (McIntyre et al., 2009). Due to the inability of Lyso-PAF to perform most of the biological functions associated with PAF C-16, this precursor analog is mostly used as a control in experiments performed with PAF C-16 (Montrucchio et al., 2000). We observed that exogenous Lyso-PAF failed to inhibit the growth of intracellular *M. smegmatis*, suggesting that the acetyl group at position *sn*-2 was important for the intracellular growth inhibitory effect of PAF C-16. Another synthetic PAF C-16 analog, 2-*O*-methyl PAF (where acetyl group of PAF C-16 at position *sn*-2 is replaced by a methyl group), was also unable to inhibit the growth of intracellular *M. smegmatis*. Structural analogs with changes in functional group at *sn*-2 position have previously been shown to lack different PAF C-16 associated activities such as aggregation of platelets (McManus et al., 1981), NO production from endothelial cells (Kikuchi et al., 2008), excitation of synaptic transmission in neuronal cells (Clark et al., 1992), and bronchial hyper-responsiveness (Cuss et al., 1986).

PAF C-18, a naturally occurring PAF C-16 analog, was also used to investigate the effect of increase in the number of carbon atoms in the aliphatic carbon tail on the intracellular *M. smegmatis* growth inhibition. PAF C-18, which has two additional carbon atoms in the aliphatic carbon tail attached at position *sn*-1 as compared to PAF C-16, was also able to inhibit the growth of intracellular *M. smegmatis*. Previous studies have shown that PAF C-18 is less potent in inducing platelet aggregation but is as potent as PAF C-16 in activating guinea pig macrophages (Stewart and Grigoriadis, 1991).

Hexanolamino PAF differs from PAF C-16 in the position of the terminal amino group, which is linked by an additional 4-carbon atoms chain to the phosphate group. This compound also inhibited the growth of intracellular *M. smegmatis*. Hexanolamino PAF has previously been shown to act as both PAF C-16 antagonist and agonist. Hexanolamino PAF can inhibit PAF C-16 stimulated production of ROI by human macrophage (Rouis et al., 1988) and act as a partial PAF C-16 agonist in guinea pig macrophages (Stewart and Grigoriadis, 1991).

To investigate the role of PAF C-16 signaling through PAFR during intracellular growth inhibition of *M. smegmatis*, PAFR antagonists were used. PAFR antagonists are compounds that can bind to PAFR and reduce PAF C-16 activity by blocking PAFR (Singh et al., 2013). Prior treatment with PAFR antagonists, ABT-491 or WEB-2086, increased the growth of intracellular *M. smegmatis* as compared to the PAF C-16 only treated condition, suggesting *M. smegmatis* intracellular growth inhibition was partly mediated through PAFR. It has also been suggested that PAF C-16 can perform certain biological activities via pathways independent of PAFR signaling (Dyer et al., 2010). PAFR antagonist, ABT-491, has been shown to be highly effective in suppressing PAF C-16 induced platelet degranulation and PAF C-16 mediated pathological conditions such as inflammation, hypotension and other lethal effects in rat and guinea pig models (Albert et al., 1997). Similarly, WEB-2086 is also a known potent PAFR antagonist, which has been shown to inhibit PAF C-16 induced activities such as platelet aggregation, hypotension and vascular permeability in rats (Clavijo et al., 2001).

Binding of PAF C-16 to PAFR results in the activation of intracellular signaling components. Therefore, we investigated the role of PAF C-16 intracellular signaling pathway components such as PLC, cPLA_2_ and different second messengers in PAF C-16 induced growth inhibition of intracellular *M. smegmatis*. Binding of PAF C-16 to PAFR on the target cell results in the activation of a membrane bound enzyme, PLC, through the associated G-proteins (Shukla, 1992). Treatment of *M. smegmatis* infected THP-1 cells with PLC inhibitor, U-73122, increased the number of surviving CFUs by 25.2%. During PAF C-16 signaling pathway, the activated PLC causes the hydrolysis of phosphatidylinositol 4,5-bisphosphate (PIP_2_) and leads to the production of second messengers, Diacylglycerol (DAG) and Inositol triphosphate (IP_3_) (Ishii and Shimizu, 2000). Since these second messengers are produced inside the cell and may come in contact with the intracellular *M. smegmatis*, we investigated PIP_2_ and DAG for their direct inhibitory effect on the growth of *M. smegmatis*. However, both these compounds did not show any direct inhibitory effect on the growth of *M. smegmatis* when tested at a range of concentrations (5, 10, 25, 50 μg and 100 μg/ml) (Supplementary Figures 3, 4), suggesting they are not directly involved in the intracellular growth inhibition activity of *M. smegmatis*.

PAF C-16 binding to PAFR also causes the activation of cytosolic phospholipase A_2_ (cPLA_2_) due to increased levels of intracellular Ca^2+^ (Ishii and Shimizu, 2000). Treatment of *M. smegmatis* infected THP-1 cells with cPLA_2_ inhibitor, Benzenesulfonamide, increased the surviving *M. smegmatis* CFUs by 30% when compared to the *M. smegmatis* infected THP-1 cells treated with PAF C-16 alone. Activated cPLA_2_ causes the production of AA from phospholipids inside the cell (Nakashima et al., 1989). In our study, AA at a concentration as low as 2.5 μg/ml (8.21 μM) significantly reduced the number of surviving *M. smegmatis* CFUs *in vitro*. This compound has previously been shown to inhibit the growth of *Staphylococcus aureus*, *Bacillus licheniformis*, and *Streptococcus pyogenes* (Raychowdhury et al., 1985; Zheng et al., 2005). AA has also been shown to be present in resting islet cells of Langerhans at a concentration of 15 μM/2000 cells, which increases to 10-fold after stimulation with carbacol (Ramanadham et al., 1992). In normal human serum, AA is present at a concentration of 500 nM/ml (Chilton et al., 1996), whereas in lungs the AA concentration reported is 1–5 μg/ml (Manku et al., 1983). Therefore, AA is available to interact with both intra and extra-cellular pathogens. Collectively, these results appear to suggest that PAF C-16 signaling via PAFR leads to the production of an intermediate intracellular signaling molecule, AA, which possesses direct growth inhibition capacity and might be involved in the growth inhibition of intracellular *M. smegmatis*. Furthermore, activation of cPLA_2_ leads to the production of Lyso-PAF from the membrane lipids inside the cell that can be converted into PAF C-16, resulting in its higher intracellular concentration. It is also known that PAF C-16 can activate its own synthesis in human monocytes (Valone, 1991). This intracellular PAF C-16 can bind to its nuclear receptors and elicit various signaling pathways resulting in the expression of proinflammatory genes such as iNOS and COX-2 (Zhu et al., 2006).

Nitric oxide (NO) and RNIs such as peroxynitrite have a damaging effect on mycobacteria (Long et al., 2005; Jamaati et al., 2017). The role of RNIs in PAF C-16 induced intracellular growth inhibition of *M. smegmatis* was investigated by using a potent iNOS inhibitor, Aminoguanidine hemisulfate (AG), which increased the survival of intracellular *M. smegmatis* by 23.4% as compared to PAF C-16 only treated condition. PAF C-16 has previously been shown to cause the production of NO and RNIs in murine cells including monocytes, macrophages and Kupffer cells through activation of iNOS enzyme (Szabo et al., 1993; Mustafa et al., 1996). NO and related nitrogen compounds have been shown to inhibit the growth of *M.tb* inside mouse macrophages (Bose et al., 1999). Furthermore, it has been shown that PAF C-16 treatment of *T. cruzi* infected mouse macrophages results in the inhibition of intracellular parasite due to PAF C-16 induced NO production (Aliberti et al., 1999).

We also observed that TNF-α neutralizing antibody (anti-TNF-α mAb) partially mitigated the growth inhibitory effect of PAF C-16 on intracellular *M. smegmatis*. PAF C-16 has previously been shown to stimulate macrophages to produce TNF-α (Dubois et al., 1989; Ruis et al., 1991). The protective role of TNF-α during *M.tb* infection is well established (Flynn et al., 1995; Keane et al., 1997; Bean et al., 1999; Mohan et al., 2001). Previous studies have shown that TNF-α is involved in the induction of NO by activation of iNOS genes in macrophages, which causes the elimination of intracellular pathogens such as *L. major* (Fonseca et al., 2003) and *T. cruzi* (Silva et al., 1995).

**FIGURE 10:**
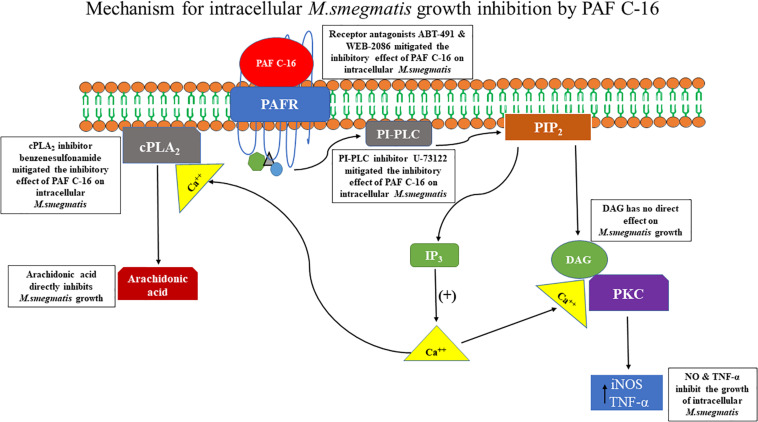
Proposed mechanism of PAF C-16 induced growth inhibition of intracellular *M. smegmatis*. Binding of PAF C-16 to its receptor PAFR on the target cell activates phosphatidylinositol specific phospholipase C (PI-PLC) enzyme which causes the production of second messengers IP_3_ and DAG inside the cell. The second messenger IP_3_ then causes the mobilization of intracellular Ca^++^. DAG along with Ca^++^ further leads to the activation of phosphokinase C (PKC) enzyme that upregulates the production of reactive nitrogen intermediates and TNF-α, which can inhibit the growth of intracellular *M. smegmatis*. In addition, the elevated level of intracellular Ca^++^ also causes the activation of cytosolic phospholipase A_2_ (cPLA_2_) enzyme that hydrolyzes phospholipids and results in the production of arachidonic acid inside the cell. This arachidonic acid has the potential to inhibit the growth of *M. smegmatis.*

In conclusion, our study shows that exogenous PAF C-16 can inhibit the intracellular growth of *M. smegmatis* involving PAFR signaling pathways, which is at least partially mediated in a NO and TNF-α dependent manner. The underlying mechanism arising from this study is summarized in the Figure 10.

## Data Availability Statement

All datasets generated for this study are included in the article/Supplementary Material.

## Author Contributions

AP and MR conceived and designed the study. AP supervised the work. MR wrote the manuscript with contributions from AP, UK, and SB. MR did the experiments with contribution from AK and SS. MR, AP, and AK analyzed the data. All authors reviewed the final version of the draft, and approved for submission.

## Conflict of Interest

The authors declare that the research was conducted in the absence of any commercial or financial relationships that could be construed as a potential conflict of interest.

## References

[B1] Alam I. Smith J. Silver M. J. (1983). Human and rabbit platelets form platelet-activating factor in response to calcium ionophore. *Thromb. Res.* 30 71–79. 10.1016/0049-3848(83)90398-5 6407140

[B2] Albert D. H. Magoc T. J. Tapang P. Luo G. Morgan D. W. Curtin M. (1997). Pharmacology of ABT-491, a highly potent platelet-activating factor receptor antagonist. *Eur. J. Pharmacol.* 325 69–80. 10.1016/s0014-2999(97)00109-x 9151941

[B3] Aliberti J. C. Machado F. S. Gazzinelli R. T. Teixeira M. M. Silva J. S. (1999). Platelet-activating factor induces nitric oxide synthesis in *Trypanosoma cruzi*-infected macrophages and mediates resistance to parasite infection in mice. *Infect. Immun.* 67 2810–2814. 10338485 10.1128/iai.67.6.2810-2814.1999 PMC96586

[B4] Amaral E. P. Lasunskaia E. B. D’Imperio-Lima M. R. (2016). Innate immunity in tuberculosis: how the sensing of mycobacteria and tissue damage modulates macrophage death. *Microbes Infect.* 18 11–20. 10.1016/j.micinf.2015.09.005 26369715

[B5] Aronson N. E. Santosham M. Comstock G. W. Howard R. S. Moulton L. H. Rhoades E. R. (2004). Long-term efficacy of BCG vaccine in American Indians and Alaska Natives: a 60-year follow-up study. *JAMA* 291 2086–2091. 10.1001/jama.291.17.2086 15126436

[B6] Bean A. G. Roach D. R. Briscoe H. France M. P. Korner H. Sedgwick J. D. (1999). Structural deficiencies in granuloma formation in TNF gene-targeted mice underlie the heightened susceptibility to aerosol *Mycobacterium tuberculosis* infection, which is not compensated for by lymphotoxin. *J. Immunol.* 162 3504–3511. 10092807

[B7] Biffl W. L. Moore E. E. Moore F. A. Barnett C. C. Silliman C. C. Peterson V. M. (1996). Interleukin-6 stimulates neutrophil production of platelet-activating factor. *J. Leukoc. Biol.* 59 569–574. 10.1002/jlb.59.4.569 8613706

[B8] Bonavida B. Mencia-Huerta J. Braquet P. (1989). Effect of platelet-activating factor on monocyte activation and production of tumor necrosis factor. *Int. Arch. Allergy Immunol.* 88 157–160. 10.1159/000234772 2707879

[B9] Borges A. F. Morato C. I. Gomes R. S. Dorta M. L. de Oliveira M. A. P. Ribeiro-Dias F. (2017). Platelet-activating factor increases reactive oxygen species-mediated microbicidal activity of human macrophages infected with *Leishmania (Viannia) braziliensis*. *Pathog. Dis.* 75:ftx082. 10.1093/femspd/ftx082 28830073

[B10] Bose M. Farnia P. Sharma S. Chattopadhya D. Saha K. (1999). Nitric oxide dependent killing of *Mycobacterium tuberculosis* by human mononuclear phagocytes from patients with active tuberculosis. *Int. J. Immunopathol. Pharmacol.* 12 69–79. 12783649

[B11] Bussolino F. Breviario F. Tetta C. Aglietta M. Mantovani A. Dejana E. (1986). Interleukin 1 stimulates platelet-activating factor production in cultured human endothelial cells. *J. Clin. Invest.* 77 2027–2033. 10.1016/0031-6989(86)90046-9 2872233 PMC370564

[B12] Camussi G. Bussolino F. Salvidio G. Baglioni C. (1987). Tumor necrosis factor/cachectin stimulates peritoneal macrophages, polymorphonuclear neutrophils, and vascular endothelial cells to synthesize and release platelet-activating factor. *J. Exp. Med.* 166 1390–1404. 10.1084/jem.166.5.1390 3119758 PMC2189646

[B13] Chanput W. Mes J. J. Wichers H. J. (2014). THP-1 cell line: an in vitro cell model for immune modulation approach. *Int. Immunopharmacol.* 23 37–45. 10.1016/j.intimp.2014.08.002 25130606

[B14] Chao W. Liu H. L. Zhou W. G. Hanahan D. J. Olson M. S. (1990). Regulation of platelet-activating factor receptor and platelet-activating factor receptor-mediated biological responses by cAMP in rat Kupffer cells. *J. Biol. Chem.* 265 17576–17583. 2170386

[B15] Chesney C. M. Pifer D. D. Byers L. W. Muirhead E. E. (1982). Effect of platelet-activating factor (PAF) on human platelets. *Blood* 59 582–585. 10.1016/0049-3848(87)90219-2 7037068

[B16] Chilton F. H. Fonteh A. N. Surette M. E. Triggiani M. Winkler J. D. (1996). Control of arachidonate levels within inflammatory cells. *Biochim. Biophys. Acta* 1299 1–15. 10.1016/0005-2760(95)00169-7 8555241

[B17] Clark G. D. Happel L. T. Zorumski C. F. Bazan N. G. (1992). Enhancement of hippocampal excitatory synaptic transmission by platelet-activating factor. *Neuron* 9 1211–1216. 10.1016/0896-6273(92)90078-r 1334422

[B18] Clavijo L. C. Carter M. B. Matheson P. J. Wilson M. A. Wead W. B. Garrison R. N. (2001). PAF increases vascular permeability without increasing pulmonary arterial pressure in the rat. *J. Appl. Physiol.* 90 261–268. 10.1152/jappl.2001.90.1.261 11133918

[B19] Clay K. L. Murphy R. C. Andres J. L. Lynch J. Henson P. M. (1984). Structure elucidation of platelet activating factor derived from human neutrophils. *Biochem. Biophys. Res. Commun.* 121 815–825. 10.1016/0006-291x(84)90751-4 6430285

[B20] Colditz G. A. Brewer T. F. Berkey C. S. Wilson M. E. Burdick E. Fineberg H. V. (1994). Efficacy of BCG vaccine in the prevention of tuberculosis: meta-analysis of the published literature. *JAMA* 271 698–702. 8309034

[B21] Cuss F. Dixon C. S. Barnes P. (1986). Effects of inhaled platelet activating factor on pulmonary function and bronchial responsiveness in man. *Lancet* 328 189–192. 10.1016/s0140-6736(86)92489-x 2873440

[B22] Daley C. L. Small P. M. Schecter G. F. Schoolnik G. K. McAdam R. A. Jacobs W. R. Jr. (1992). An outbreak of tuberculosis with accelerated progression among persons infected with the human immunodeficiency virus: an analysis using restriction-fragment—length polymorphisms. *N. Engl. J. Med.* 326 231–235. 10.1056/NEJM199201233260404 1345800

[B23] Demopoulos C. A. Pinckard R. N. Hanahan D. J. (1979). Platelet-activating factor. Evidence for 1-O-alkyl-2-acetyl-sn-glyceryl-3-phosphorylcholine as the active component (a new class of lipid chemical mediators). *J. Biol. Chem.* 254 9355–9358. 489536

[B24] Dubois C. Bissonnette E. Rola-Pleszczynski M. (1989). Platelet-activating factor (PAF) enhances tumor necrosis factor production by alveolar macrophages. Prevention by PAF receptor antagonists and lipoxygenase inhibitors. *J. Immunol.* 143 964–970. 2545780

[B25] Dyer K. D. Percopo C. M. Xie Z. Yang Z. Kim J. D. Davoine F. (2010). Mouse and human eosinophils degranulate in response to platelet-activating factor (PAF) and lysoPAF via a PAF-receptor-independent mechanism: evidence for a novel receptor. *J. Immunol.* 184 6327–6334. 10.4049/jimmunol.0904043 20421642 PMC3406327

[B26] Espinal M. A. Kim S. J. Suarez P. G. Kam K. M. Khomenko A. G. Migliori G. B. (2000). Standard short-course chemotherapy for drug-resistant tuberculosis: treatment outcomes in 6 countries. *JAMA* 283 2537–2545. 10.1001/jama.283.19.2537 10815117

[B27] Farooqui A. A. Ong W. Y. Horrocks L. A. (2006). Inhibitors of brain phospholipase A2 activity: their neuropharmacological effects and therapeutic importance for the treatment of neurologic disorders. *Pharmacol. Rev.* 58 591–620. 10.1124/pr.58.3.7 16968951

[B28] Flynn J. L. Chan J. (2001). Tuberculosis: latency and reactivation. *Infect. Immun.* 69 4195–4201. 11401954 10.1128/IAI.69.7.4195-4201.2001 PMC98451

[B29] Flynn J. L. Goldstein M. M. Chan J. Triebold K. J. Pfeffer K. Lowenstein C. J. (1995). Tumor necrosis factor-α is required in the protective immune response against *Mycobacterium tuberculosis* in mice. *Immunity* 2 561–572. 10.1016/1074-7613(95)90001-2 7540941

[B30] Fonseca S. G. Romão P. R. Figueiredo F. Morais R. H. Lima H. C. Ferreira S. H. (2003). TNF-α mediates the induction of nitric oxide synthase in macrophages but not in neutrophils in experimental cutaneous leishmaniasis. *Eur. J. Immunol.* 33 2297–2306. 10.1002/eji.200320335 12884305

[B31] Fontán P. Aris V. Ghanny S. Soteropoulos P. Smith I. (2008). Global transcriptional profile of *Mycobacterium tuberculosis* during THP-1 human macrophage infection. *Infect. Immun.* 76 717–725. 10.1128/IAI.00974-07 18070897 PMC2223452

[B32] Gawaz M. Langer H. May A. E. (2005). Platelets in inflammation and atherogenesis. *J. Clin. Invest.* 115 3378–3384. 16322783 10.1172/JCI27196 PMC1297269

[B33] Hartung H. Parnham M. J. Winkelmann J. Englberger W. Hadding U. (1983). Platelet activating factor (PAF) induces the oxidative burst in macrophages. *Int. J. Immunopharmacol.* 5 115–121. 10.1016/0192-0561(83)90002-4 6874164

[B34] Henderson W. R. Jr. Lu J. Poole K. M. Dietsch G. N. Chi E. Y. (2000). Recombinant human platelet-activating factor-acetylhydrolase inhibits airway inflammation and hyperreactivity in mouse asthma model. *J. Immunol.* 164 3360–3367. 10.4049/jimmunol.164.6.3360 10706731

[B35] Honda Z. Ishii S. Shimizu T. (2002). Platelet-activating factor receptor. *J. Biochem.* 131 773–779. 12038971 10.1093/oxfordjournals.jbchem.a003164

[B36] Huseyinov A. Kutukculer N. Aydogdu S. Caglayan S. Coker I. Goksen D. (1999). Increased gastric production of platelet-activating factor, leukotriene-B4, and tumor necrosis factor-α in children with *Helicobacter pylori* infection. *Dig. Dis. Sci.* 44 675–679. 10.1023/a:1012941020790 10219821

[B37] Im S. Y. Choi J. H. Ko H. M. Han S. J. Chun S. B. Lee H. K. (1997). A protective role of platelet-activating factor in murine candidiasis. *Infect. Immun.* 65 1321–1326. 9119469 10.1128/iai.65.4.1321-1326.1997 PMC175135

[B38] Iona E. Pardini M. Gagliardi M. C. Colone M. Stringaro A. R. Teloni R. (2012). Infection of human THP-1 cells with dormant *Mycobacterium tuberculosis*. *Microbes Infect.* 14 959–967. 10.1016/j.micinf.2012.04.003 22546526

[B39] Ishii S. Nagase T. Shimizu T. (2002). Platelet-activating factor receptor. *Prostaglandins Other Lipid Mediat.* 68 599–609. 12432946 10.1016/s0090-6980(02)00058-8

[B40] Ishii S. Shimizu T. (2000). Platelet-activating factor (PAF) receptor and genetically engineered PAF receptor mutant mice. *Prog. Lipid Res.* 39 41–82. 10.1016/s0163-7827(99)00016-8 10729607

[B41] Jamaati H. Mortaz E. Pajouhi Z. Folkerts G. Movassaghi M. Moloudizargari M. (2017). Nitric oxide in the pathogenesis and treatment of tuberculosis. *Front. Microbiol.* 8:2008. 10.3389/fmicb.2017.02008 29085351 PMC5649180

[B42] Kaminskaia G. O. Aladysheva Z. (1995). Platelet activating factor and pulmonary tuberculosis. *Vestn. Ross. Akad. Med. Nauk* 7 45–48. 7670343

[B43] Kaul M. Loos M. (1995). Collagen-like complement component C1q is a membrane protein of human monocyte-derived macrophages that mediates endocytosis. *J. Immunol.* 155 5795–5802. 7499868

[B44] Keane J. Balcewicz-Sablinska M. K. Remold H. G. Chupp G. L. Meek B. B. Fenton M. J. (1997). Infection by *Mycobacterium tuberculosis* promotes human alveolar macrophage apoptosis. *Infect. Immun.* 65 298–304. 8975927 10.1128/iai.65.1.298-304.1997 PMC174591

[B45] Kikuchi M. Shirasaki H. Himi T. (2008). Platelet-activating factor (PAF) increases NO production in human endothelial cells—real-time monitoring by DAR-4M AM. *Prostaglandins Leukot. Essent. Fatty Acids* 78 305–309. 10.1016/j.plefa.2008.04.002 18502110

[B46] Kim H. Kim S. Ko H. Choi J. Kim K. Oh S. (2008). Nitric oxide plays a key role in the platelet-activating factor-induced enhancement of resistance against systemic candidiasis. *Immunology* 124 428–435. 10.1111/j.1365-2567.2007.02795.x 18397269 PMC2440837

[B47] Lahey T. von Reyn C. F. (2016). *Mycobacterium bovis* BCG and new vaccines for the prevention of tuberculosis. *Microbiol. Spectr.* 4 187–209. 10.1128/microbiolspec.TNMI7-0003-2016 27763257

[B48] Leaver H. Qu J. Smith G. Howie A. Ross W. Yap P. (1990). Endotoxin releases platelet-activating factor from human monocytes in vitro. *Immunopharmacology* 20 105–113. 10.1016/0162-3109(90)90013-5 2266000

[B49] Leemans J. C. Thepen T. Weijer S. Florquin S. Van Rooijen N. Van de Winkel J. G. (2005). Macrophages play a dual role during pulmonary tuberculosis in mice. *J. Infect. Dis.* 191 65–74. 10.1086/426395 15593005

[B50] Liu C. H. Li L. Chen Z. Wang Q. Hu Y. L. Zhu B. (2011). Characteristics and treatment outcomes of patients with MDR and XDR tuberculosis in a TB referral hospital in Beijing: a 13-year experience. *PLoS One* 6:e19399. 10.1371/journal.pone.0019399 21559362 PMC3084844

[B51] Lonardoni M. V. Russo M. Jancar S. (2000). Essential role of platelet-activating factor in control of *Leishmania (Leishmania) amazonensis* infection. *Infect. Immun.* 68 6355–6361. 10.1128/iai.68.11.6355-6361.2000 11035745 PMC97719

[B52] Long R. Jones R. Talbot J. Mayers I. Barrie J. Hoskinson M. (2005). Inhaled nitric oxide treatment of patients with pulmonary tuberculosis evidenced by positive sputum smears. *Antimicrob. Agents Chemother.* 49 1209–1212. 10.1128/AAC.49.3.1209-1212.2005 15728930 PMC549277

[B53] Macmillan D. McCarron J. (2010). The phospholipase C inhibitor U-73122 inhibits Ca2 release from the intracellular sarcoplasmic reticulum Ca2 store by inhibiting Ca2 pumps in smooth muscle. *Br. J. Pharmacol.* 160 1295–1301. 10.1111/j.1476-5381.2010.00771.x 20590621 PMC2938802

[B54] Manku M. Horrobin D. Huang Y. Morse N. (1983). Fatty acids in plasma and red cell membranes in normal humans. *Lipids* 18 906–908. 10.1007/BF02534572 6664259

[B55] McIntyre T. M. Prescott S. M. Stafforini D. M. (2009). The emerging roles of PAF acetylhydrolase. *J. Lipid Res.* 50(Suppl.), S255–S259. 18838739 10.1194/jlr.R800024-JLR200 PMC2674695

[B56] McManus L. M. Hanahan D. J. Pinckard R. N. (1981). Human platelet stimulation by acetyl glyceryl ether phosphorylcholine. *J. Clin. Invest.* 67 903–906. 10.1172/jci110108 7204562 PMC370642

[B57] Mohan V. P. Scanga C. A. Yu K. Scott H. M. Tanaka K. E. Tsang E. (2001). Effects of tumor necrosis factor alpha on host immune response in chronic persistent tuberculosis: possible role for limiting pathology. *Infect. Immun.* 69 1847–1855. 10.1128/IAI.69.3.1847-1855.2001 11179363 PMC98092

[B58] Montrucchio G. Alloatti G. Camussi G. (2000). Role of platelet-activating factor in cardiovascular pathophysiology. *Physiol. Rev.* 80 1669–1699. 10.1152/physrev.2000.80.4.1669 11015622

[B59] Muehlmann L. A. Michelotto P. V. Jr. Nunes E. A. Grando F. C. C. da Silva F. T. Nishiyama A. (2012). PAF increases phagocytic capacity and superoxide anion production in equine alveolar macrophages and blood neutrophils. *Res. Vet. Sci.* 93 393–397. 10.1016/j.rvsc.2011.07.008 21820686

[B60] Mustafa S. B. Howard K. M. Olson M. S. (1996). Platelet-activating factor augments lipopolysaccharide-induced nitric oxide formation by rat Kupffer cells. *Hepatology* 23 1622–1630. 10.1002/hep.510230645 8675186

[B61] Nakashima S. Suganuma A. Sato M. Tohmatsu T. Nozawa Y. (1989). Mechanism of arachidonic acid liberation in platelet-activating factor-stimulated human polymorphonuclear neutrophils. *J. Immunol.* 143 1295–1302. 2545786

[B62] Nascimento F. R. Calich V. L. Rodriguez D. Russo M. (2002). Dual role for nitric oxide in paracoccidioidomycosis: essential for resistance, but overproduction associated with susceptibility. *J. Immunol.* 168 4593–4600. 10.4049/jimmunol.168.9.4593 11971007

[B63] O’Flaherty J. T. Redman J. Jr. Schmitt J. D. Ellis J. M. Surles J. R. Marx M. H. (1987). 1-0-alkyl-2-N-methylcarbamyl-glycerophosphocholine: a biologically potent, non-metabolizable analog of platelet-activating factor. *Biochem. Biophys. Res. Commun.* 147 18–24. 10.1016/s0006-291x(87)80081-5 2820395

[B64] Ormerod L. P. (2005). Multidrug-resistant tuberculosis (MDR-TB): epidemiology, prevention and treatment. *Br. Med. Bull.* 73 17–24. 10.1093/bmb/ldh047 15956357

[B65] Poubelle P. E. Gingras D. Demers C. Dubois C. Harbour D. Grassi J. (1991). Platelet-activating factor (PAF-acether) enhances the concomitant production of tumour necrosis factor-alpha and interleukin-1 by subsets of human monocytes. *Immunology* 72 181–187. 2016118 PMC1384481

[B66] Pustynnikov M. Porodenko N. Makarova O. Kozyukov A. Moskaleva E. Y. Sokolovsky A. (1991). Platelet-activating factor stimulates receptor-mediated fromation of reactive oxygen intermediates in human monocytes. *Lipids* 26 1214–1217. 10.1007/BF02536534 1668118

[B67] Rajavelu P. Das S. D. (2007). A correlation between phagocytosis and apoptosis in THP-1 cells infected with prevalent strains of *Mycobacterium tuberculosis*. *Microbiol. Immunol.* 51 201–210. 10.1111/j.1348-0421.2007.tb03902.x 17310088

[B68] Ramanadham S. Gross R. Turk J. (1992). Arachidonic acid induces an increase in the cytosolic calcium concentration in single pancreatic islet beta cells. *Biochem. Biophys. Res. Commun.* 184 647–653. 10.1016/0006-291x(92)90638-2 1575739

[B69] Raychowdhury M. Goswami R. Chakrabarti P. (1985). Effect of unsaturated fatty acids in growth inhibition of some penicillin-resistant and sensitive bacteria. *J. Appl. Bacteriol.* 59 183–188. 10.1111/j.1365-2672.1985.tb03319.x 3900022

[B70] Riaz M. S. Kaur A. Shwayat S. N. Behboudi S. Kishore U. Pathan A. A. (2018). Direct growth inhibitory effect of platelet activating factor C-16 and its structural analogs on mycobacteria. *Front. Microbiol.* 9:1903. 10.3389/fmicb.2018.01903 30258409 PMC6143801

[B71] Richter E. Ventz K. Harms M. Mostertz J. Hochgräfe F. (2016). Induction of macrophage function in human THP-1 cells is associated with rewiring of MAPK signaling and activation of MAP3K7 (TAK1) protein kinase. *Front. Cell Dev. Biol.* 4:21. 10.3389/fcell.2016.00021 27066479 PMC4811913

[B72] Riendeau C. J. Kornfeld H. (2003). THP-1 cell apoptosis in response to Mycobacterial infection. *Infect. Immun.* 71 254–259. 10.1128/iai.71.1.254-259.2003 12496173 PMC143334

[B73] Rohan D. Mahesh K. Manoj R. Sekhar M. (2008). Inhibition of bfl-1/A1 by siRNA inhibits mycobacterial growth in THP-1 cells by enhancing phagosomal acidification. *Biochim. Biophys. Acta* 1780 733–742. 10.1016/j.bbagen.2007.12.010 18206119

[B74] Rose J. K. Debs R. A. Philip R. Ruis N. M. Valone F. H. (1990). Selective activation of human monocytes by the platelet-activating factor analog 1-O-hexadecyl-2-O-methyl-sn-glycero-3-phosphorylcholine. *J. Immunol.* 144 3513–3517. 2329280

[B75] Rouis M. Nigon F. Chapman M. J. (1988). Platelet activating factor is a potent stimulant of the production of active oxygen species by human monocyte-derived macrophages. *Biochem. Biophys. Res. Commun.* 156 1293–1301. 10.1016/s0006-291x(88)80773-3 2847729

[B76] Ruis N. Rose J. Valone F. (1991). Tumor necrosis factor release by human monocytes stimulated with platelet-activating factor. *Lipids* 26 1060–1064. 10.1007/BF02536502 1668105

[B77] Schleimer R. P. MacGlashan D. W. Jr. Peters S. P. Pinckard R. N. Adkinson N. F. Jr. Lichtenstein L. M. (1986). Characterization of inflammatory mediator release from purified human lung mast cells. *Am. Rev. Respir. Dis.* 133 614–617. 10.1164/arrd.1986.133.4.614 3485946

[B78] Sepulveda R. Parcha C. Sorensen R. (1992). Case-control study of the efficacy of BCG immunization against pulmonary tuberculosis in young adults in Santiago, Chile. *Tuber. Lung Dis.* 73 372–377. 10.1016/0962-8479(92)90043-J 1292719

[B79] Sherwood E. R. Toliver-Kinsky T. (2004). Mechanisms of the inflammatory response. *Best Pract. Res. Clin. Anaesthesiol.* 18 385–405. 15212335 10.1016/j.bpa.2003.12.002

[B80] Shigenobu K. Masuda Y. Tanaka Y. Kasuya T. (1985). Platelet activating factor analogues: lack of correlation between their activities to produce hypotension and endothelium-mediated vasodilation. *J. Pharmacobiodyn* 8 128–133. 10.1248/bpb1978.8.128 4009403

[B81] Shukla S. D. (1992). Platelet-activating factor receptor and signal transduction mechanisms. *FASEB J.* 6 2296–2301. 10.1096/fasebj.6.6.1312046 1312046

[B82] Silva J. S. Vespa G. N. Cardoso M. A. Aliberti J. C. Cunha F. Q. (1995). Tumor necrosis factor alpha mediates resistance to *Trypanosoma cruzi* infection in mice by inducing nitric oxide production in infected gamma interferon-activated macrophages. *Infect. Immun.* 63 4862–4867. 7591147 10.1128/iai.63.12.4862-4867.1995 PMC173696

[B83] Simon H. U. Tsao P. W. Siminovitch K. A. Mills G. B. Blaser K. (1994). Functional platelet-activating factor receptors are expressed by monocytes and granulocytes but not by resting or activated T and B lymphocytes from normal individuals or patients with asthma. *J. Immunol.* 153 364–377. 8207248

[B84] Singh P. Singh I. N. Mondal S. C. Singh L. Garg V. K. (2013). Platelet-activating factor (PAF)-antagonists of natural origin. *Fitoterapia* 84 180–201. 10.1016/j.fitote.2012.11.002 23160091

[B85] Steel H. Cockeran R. Anderson R. (2002). Platelet-activating factor and lyso-PAF possess direct antimicrobial properties in vitro. *APMIS* 110 158–164. 10.1034/j.1600-0463.2002.100206.x 12064871

[B86] Stewart A. G. Grigoriadis G. (1991). Structure-activity relationships for platelet-activating factor (PAF) and analogues reveal differences between PAF receptors on platelets and macrophages. *J. Lipid Mediat.* 4 299–308. 1662547

[B87] Szabo C. Wu C. C. Mitchell J. A. Gross S. S. Thiemermann C. Vane J. R. (1993). Platelet-activating factor contributes to the induction of nitric oxide synthase by bacterial lipopolysaccharide. *Circ. Res.* 73 991–999. 10.1161/01.res.73.6.991 7693362

[B88] Vadas P. Perelman B. Liss G. (2013). Platelet-activating factor, histamine, and tryptase levels in human anaphylaxis. *J. Allergy Clin. Immunol.* 131 144–149. 10.1016/j.jaci.2012.08.016 23040367

[B89] Valone F. H. (1991). Synthesis of platelet-activating factor by human monocytes stimulated by platelet-activating factor. *J. Allergy Clin. Immunol.* 87 715–720. 2005325 10.1016/0091-6749(91)90394-4

[B90] Verouti S. N. Fragopoulou E. Karantonis H. C. Dimitriou A. A. Tselepis A. D. Antonopoulou S. (2011). PAF effects on MCP-1 and IL-6 secretion in U-937 monocytes in comparison with OxLDL and IL-1β effects. *Atherosclerosis* 219 519–525. 10.1016/j.atherosclerosis.2011.07.123 21920519

[B91] Vlachogianni I. C. Nomikos T. Fragopoulou E. Stamatakis G. M. Karantonis H. C. Antonopoulou S. (2013). Interleukin-1beta stimulates platelet-activating factor production in U-937 cells modulating both its biosynthetic and catabolic enzymes. *Cytokine* 63 97–104. 10.1016/j.cyto.2013.04.024 23673285

[B92] Weijer S. Leemans J. C. Florquin S. Shimizu T. Ishii S. Van Der Poll T. (2003). Host response of platelet-activating factor receptor-deficient mice during pulmonary tuberculosis. *Immunology* 109 552–556. 10.1046/j.1365-2567.2003.01688.x 12871222 PMC1783009

[B93] World Health Organization [WHO] (2018). *Global Tuberculosis Report 2018.* Geneva: World Health Organization.

[B94] Yagnik D. (2014). Macrophage derived platelet activating factor implicated in the resolution phase of gouty inflammation. *Int. J. Inflam.* 2014:526496. 10.1155/2014/526496 25328755 PMC4190697

[B95] Zheng C. J. Yoo J. Lee T. Cho H. Kim Y. Kim W. (2005). Fatty acid synthesis is a target for antibacterial activity of unsaturated fatty acids. *FEBS Lett.* 579 5157–5162. 10.1016/j.febslet.2005.08.028 16146629

[B96] Zhu T. Gobeil F. Vazquez-Tello A. Leduc M. Rihakova L. Bossolasco M. (2006). Intracrine signaling through lipid mediators and their cognate nuclear G-protein-coupled receptors: a paradigm based on PGE2, PAF, and LPA1 receptors. *Can. J. Physiol. Pharmacol.* 84 377–391. 10.1139/y05-147 16902584

