# Supplementary material for: Dissecting the Mechanism of Intracellular Mycobacterium smegmatis Growth Inhibition by Platelet Activating Factor C-16
Source: Front Microbiol. 2020 Jun 10;11:1046. doi: 10.3389/fmicb.2020.01046 (PMC7297918; doi:10.3389/fmicb.2020.01046)
Supplement: Supplementary file 1 [file Data_Sheet_1.docx]

**Supplementary Data**


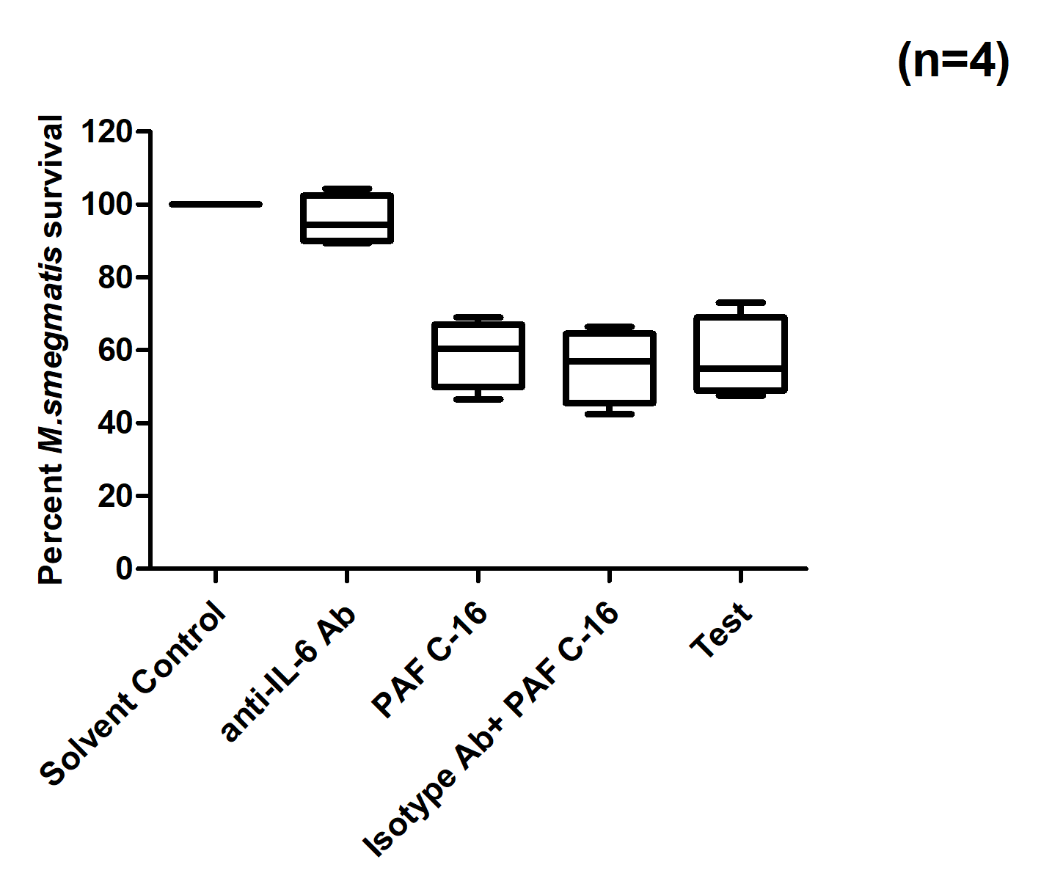


**Figure 1: Effect of anti-IL-6 neutralizing antibody on PAF C-16 induced intracellular *M.smegmatis* growth inhibition.** *M.smegmatis* infected THP1 cells were treated for 24 hours either with solvent control (10µl ethanol/ml), anti-IL-6 antibody (10µg/ml), PAF C-16 (1µg/ml), a combination of isotype control antibody (10µg/ml) with PAF C-16 (1µg/ml) and anti-IL-6 antibody (10µg/ml) with PAF C-16 (1µg/ml) (Test), before lysis and plating. The data is expressed in terms of percentage, where solvent control is considered as 100% survival and different treatment conditions are compared to it. The data represent median, interquartile and minimal and maximal values for four individual experiments performed in triplicates. Statistically non-significant differences were found for ‘Test’ v 1µg/ml PAF C-16 and ‘Test’ v Isotype control condition using Mann Whitney test.


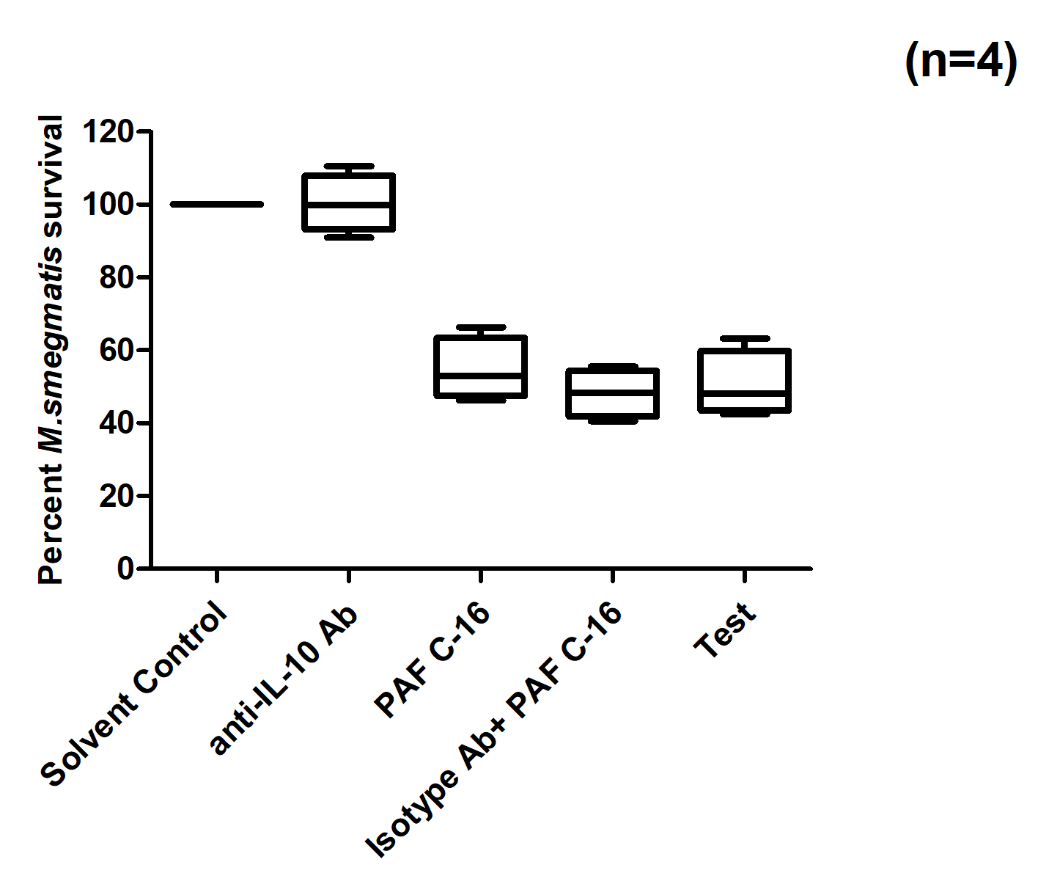


**Figure 2: Effect of anti-IL-10 neutralizing antibody on PAF C-16 induced intracellular *M.smegmatis* growth inhibition.** *M.smegmatis* infected THP-1 cells were treated for 24 hours either with solvent control (10µl ethanol/ml), anti-IL-10 antibody (10µg/ml), PAF C-16 (1µg/ml), a combination of isotype control antibody (10µg/ml) with PAF C-16 (1µg/ml) and anti-IL-10 antibody (10µg/ml) with PAF C-16 (1µg/ml) (Test), before lysis and plating. The data is expressed in terms of percentage, where solvent control is considered as 100% survival and different treatment conditions are compared to it. The data represent median, interquartile and minimal and maximal values for four individual experiments performed in triplicates. Statistically non-significant differences were found for ‘Test’ v 1µg/ml PAF C-16 and ‘Test’ v Isotype control condition using Mann Whitney test.


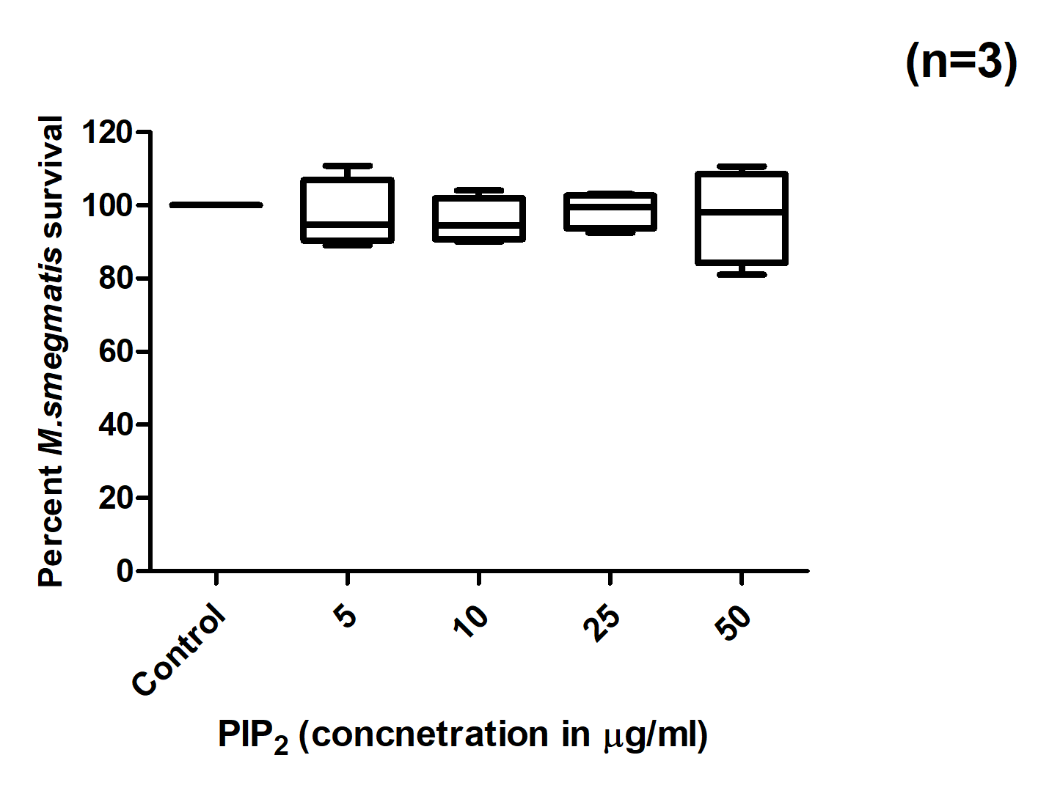


**Figure 3: Direct effect of Phosphoinositol bisphosphate (PIP_2_) on *M.smegmatis* survival *in vitro*.** *M. smegmatis* samples were treated either with solvent control (10µl ethanol/ml) or indicated concentrations of PIP_2_ for 2 hours before plating. Data is expressed in percentage where solvent control is taken as 100% survival and different PIP_2_ treated conditions are compared to it. . The data is expressed in terms of percentage, where solvent control is considered as 100% survival and different treatment conditions are compared to it. The data represent median, interquartile and minimal and maximal values for four individual experiments performed in triplicates. Statistically significant differences between PIP_2_ treated and solvent control samples were estimated using Kruskal-Wallis test and the results were found to be non-significant.


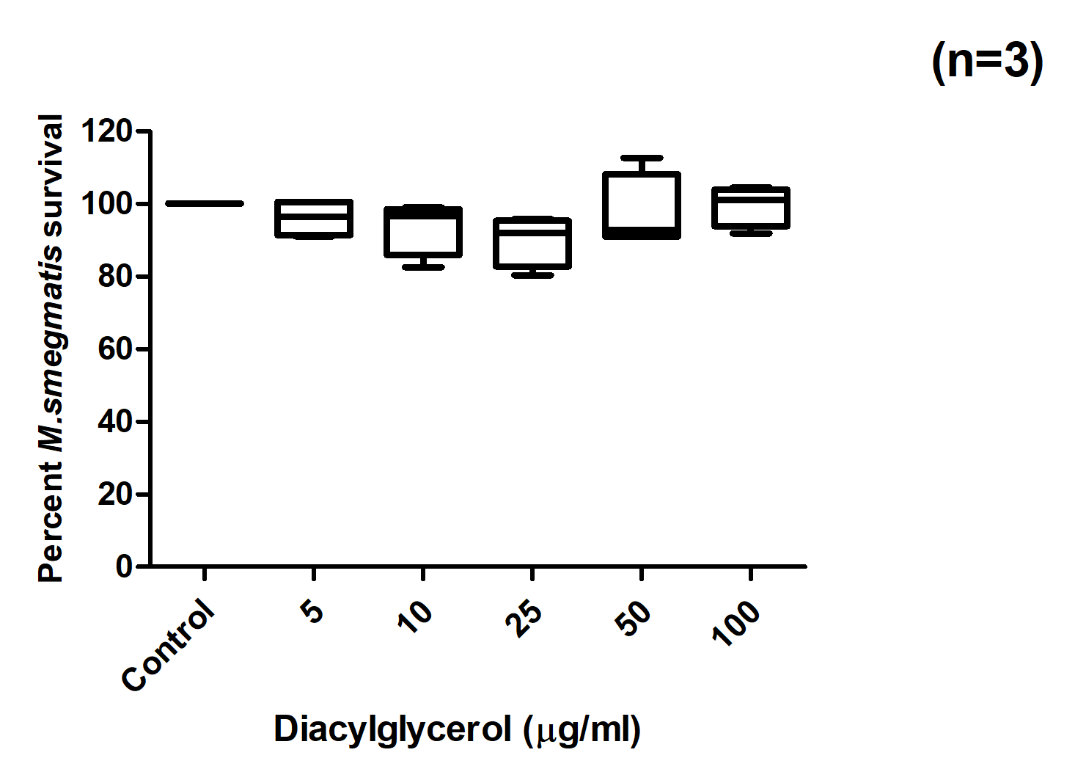


**Figure 4: Direct effect of Diacylglycerol (DAG) on *M.smegmatis* survival *in vitro*.** *M. smegmatis* samples were treated either with solvent control (10µl ethanol/ml) or indicated concentrations of DAG for 2 hours before plating. The data is expressed in terms of percentage, where solvent control is considered as 100% survival and different treatment conditions are compared to it. The data represent median, interquartile and minimal and maximal values for four individual experiments performed in triplicates. Statistically significant differences between DAG treated and solvent control samples were estimated using Kruskal-Wallis test and the results were found to be non-significant.
